# Absence of microglia promotes diverse pathologies and early lethality in Alzheimer’s disease mice

**DOI:** 10.1016/j.celrep.2022.110961

**Published:** 2022-06-14

**Authors:** Sepideh Kiani Shabestari, Samuel Morabito, Emma Pascal Danhash, Amanda McQuade, Jessica Ramirez Sanchez, Emily Miyoshi, Jean Paul Chadarevian, Christel Claes, Morgan Alexandra Coburn, Jonathan Hasselmann, Jorge Hidalgo, Kayla Nhi Tran, Alessandra C. Martini, Winston Chang Rothermich, Jesse Pascual, Elizabeth Head, David A. Hume, Clare Pridans, Hayk Davtyan, Vivek Swarup, Mathew Blurton-Jones

**Affiliations:** 1 Department of Neurobiology & Behavior, UC Irvine, Irvine, CA 92697, USA; 2 Sue and Bill Gross Stem Cell Research Center, UC Irvine, Irvine, CA 92697, USA; 3 Mathematical, Computational and System Biology (MCSB) Program, UC Irvine, Irvine, CA 92697, USA; 4 Institute for Memory Impairments and Neurological Disorders, UC Irvine, Irvine, CA 92697, USA; 5 Department of Pathology & Laboratory Medicine, UC Irvine, Irvine, CA 92697, USA; 6 Mater Research Institute-University of Queensland, Brisbane, Australia; 7 University of Edinburgh Centre for Inflammation Research, Edinburgh, UK; 8 Simons Initiative for the Developing Brain Centre, University of Edinburgh, Edinburgh, UK; 9 The Muir Maxwell Epilepsy Centre, University of Edinburgh, Edinburgh, UK; 10 Lead contact

## Abstract

Microglia are strongly implicated in the development and progression of Alzheimer’s disease (AD), yet their impact on pathology and lifespan remains unclear. Here we utilize a CSF1R hypomorphic mouse to generate a model of AD that genetically lacks microglia. The resulting microglial-deficient mice exhibit a profound shift from parenchymal amyloid plaques to cerebral amyloid angiopathy (CAA), which is accompanied by numerous transcriptional changes, greatly increased brain calcification and hemorrhages, and premature lethality. Remarkably, a single injection of wild-type microglia into adult mice repopulates the microglial niche and prevents each of these pathological changes. Taken together, these results indicate the protective functions of microglia in reducing CAA, blood-brain barrier dysfunction, and brain calcification. To further understand the clinical implications of these findings, human AD tissue and iPSC-microglia were examined, providing evidence that microglia phagocytose calcium crystals, and this process is impaired by loss of the AD risk gene, TREM2.

## In brief

Kiani Shabestari et al. show that genetic absence of microglia in Alzheimer’s disease mice causes a shift from parenchymal amyloid plaques to cerebral amyloid angiopathy, brain calcification and hemorrhages, and premature lethality. Adult microglial transplantation rescues these pathological changes, demonstrating that microglia protect the brain against detrimental Alzheimer’s disease co-pathologies.

## Graphical abstract

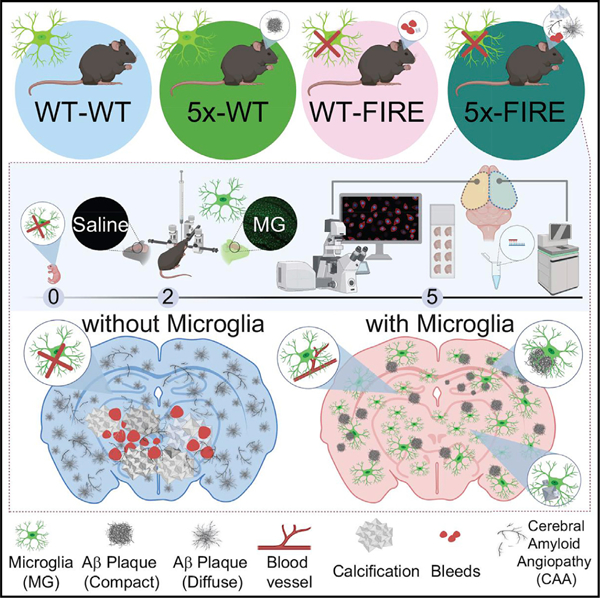

## INTRODUCTION

Alzheimer’s disease (AD) is the leading cause of age-related dementia and increasingly recognized as a neuroimmune disorder. As brain-resident immune cells, microglia are implicated in both the genetics and pathology of AD. For example, neuropathological studies have for decades described the juxtaposition of microglia around beta-amyloid (Aβ) plaques. More recently, genome-wide association studies (GWASs) have identified over 50 AD-linked loci (Jansen et al., 2019; Kunkle et al., 2019; Schwartzentruber et al., 2021), the great majority of which are associated with genes that are highly or even exclusively expressed by microglia (Efthymiou and Goate, 2017; McQuade and Blurton-Jones, 2019). Studies in AD mouse models have further identified a disease-associated microglial (DAM) expression signature within plaque-associated microglia and shown that AD risk genes alter the ability of microglia to respond to plaques and induce a DAM transcriptomic profile (Jay et al., 2017; Keren-Shaul et al., 2017; McQuade et al., 2020; Yuan et al., 2016). Collectively, these studies indicate that the microglial DAM response likely protects against amyloid-induced neuronal damage.

The contribution of microglia to AD has been examined in many animal models by pharmacological depletion or deletion of microglial genes. The impacts of these interventions on plaque deposition appear to depend upon age, disease state, and microglial gene expression. For example, pharmacological depletion of microglia at early ages decreases amyloid plaque load but at later ages has no effect on amyloid pathology (Sosna et al., 2018; Spangenberg et al., 2016, 2019). Deletion of the AD risk gene, triggering receptor expressed on myeloid cells 2 (*TREM2*), similarly decreased the initial deposition of amyloid but conversely promotes amyloid deposition at later ages (Jay et al., 2017). Reduced microglial phagocytosis of Aβ via deletion of *Mer* and *Axl* receptors was also recently shown to diminish dense core plaque pathology (Huang et al., 2021). Pharmacological microglial depletion also modulates several other AD-associated phenotypes. For example, depletion of microglia in 9-month-old 5xfAD mice reduces synaptic and neuronal loss and improves cognition, despite having no effect on Aβ plaques (Spangenberg et al., 2016). Yet other studies have found that microglial depletion reduces tau-associated neurodegeneration (Shi et al., 2019). Collectively, these experiments have clarified the impact of microglia on Aβ plaque load, neurodegeneration, and cognitive function. However, far fewer studies have examined the potential role of microglia in one of the most prevalent AD co-pathologies, cerebral amyloid angiopathy (CAA). Yet, up to 48% of AD patients exhibit substantial CAA, which involves the accumulation of insoluble Aβ within small and medium blood vessels of the brain and meninges (Jäkel et al., 2021). This vascular pathology in turn impairs the localized regulation of oxygen and glucose supply to the neurovascular unit, promoting cerebral hypoperfusion and microhemorrhages (Bell and Zlokovic, 2009). Importantly, CAA is also associated with a more rapid onset of dementia, diminished episodic memory, and increased mortality even when controlling for plaque and tangle loads (Arvanitakis et al., 2011; Vidoni et al., 2016). Given the prevalence of CAA in AD and its impact on cognition and mortality, AD risk genes likely also contribute to the development of this important pathology. Indeed, apolipoprotein 4 (APOE4), the strongest genetic risk factor for AD, increases the accumulation of Aβ within cerebral vessels, promoting the development of CAA (Tai et al., 2016).

To further understand the impact of microglia on AD-associated pathologies we crossed 5xfAD transgenic mice (Oakley et al., 2006) with FIRE mice, a mouse model that is entirely microglia-deficient but retains meningeal and perivascular macrophages (Rojo et al., 2019). The FIRE mice harbor a specific deletion of the microglial-associated *fms* intronic regulatory element (FIRE) enhancer of *Csf1r* locus to produce mice that lack microglia but retain peripheral macrophage populations. Importantly, FIRE mice do not exhibit hydrocephalus and other neurological impacts or the severe peripheral growth and developmental abnormalities reported in constitutive *Csf1r*^−/−^ rodents and humans (Hume et al., 2020; Patkar et al., 2021; Rojo et al., 2019). This model of microglial deficiency also avoids the confounding impacts of CSF1R antagonists, which drive widespread microglial apoptosis, leading to pleiotropic effects on astrocyte function and T cell recruitment (Elmore et al., 2014; Marino Lee et al., 2021; Unger et al., 2018).

In the absence of microglia, we found that Aβ neuropathology undergoes a marked shift toward vascular deposition. Using single-nucleus RNA-seq (snRNA-seq), we performed an unbiased transcriptomic analysis of 5x-FIRE and 5xfAD mice and found substantial alterations in the gene expression landscape in neuronal, glial, and brain vascular cell types. Furthermore, we found that 5x-FIRE mice develop robust brain calcification, cerebral hemorrhages, and premature death compared with 5xfAD littermates. Importantly, a single adult bilateral injection of donor microglia restored occupancy of the microglial niche and completely reversed the previously observed changes in amyloid pathology, brain calcification, and cerebral hemorrhage. In addition, snRNA-seq analysis demonstrated that many of the transcriptional defects associated with a lack of microglia in 5xfAD mice were rescued across cell types following microglial transplantation. To further explore the translational implications of these findings, we examined human AD patient brain samples and *TREM2* knockout human microglia. These studies revealed a significant increase in brain calcification in AD patients who exhibit vascular pathology and further show that *TREM2* is necessary for efficient phagocytosis of calcium crystals. Taken together, these data demonstrate that microglia regulate vascular health, calcification, and mortality in the context of amyloid pathology and further suggest that microglial AD risk genes likely influence the development of these diverse pathologies.

## RESULTS

### FIRE mice provide a genetic approach to model microglial absence

**Figure 1.**
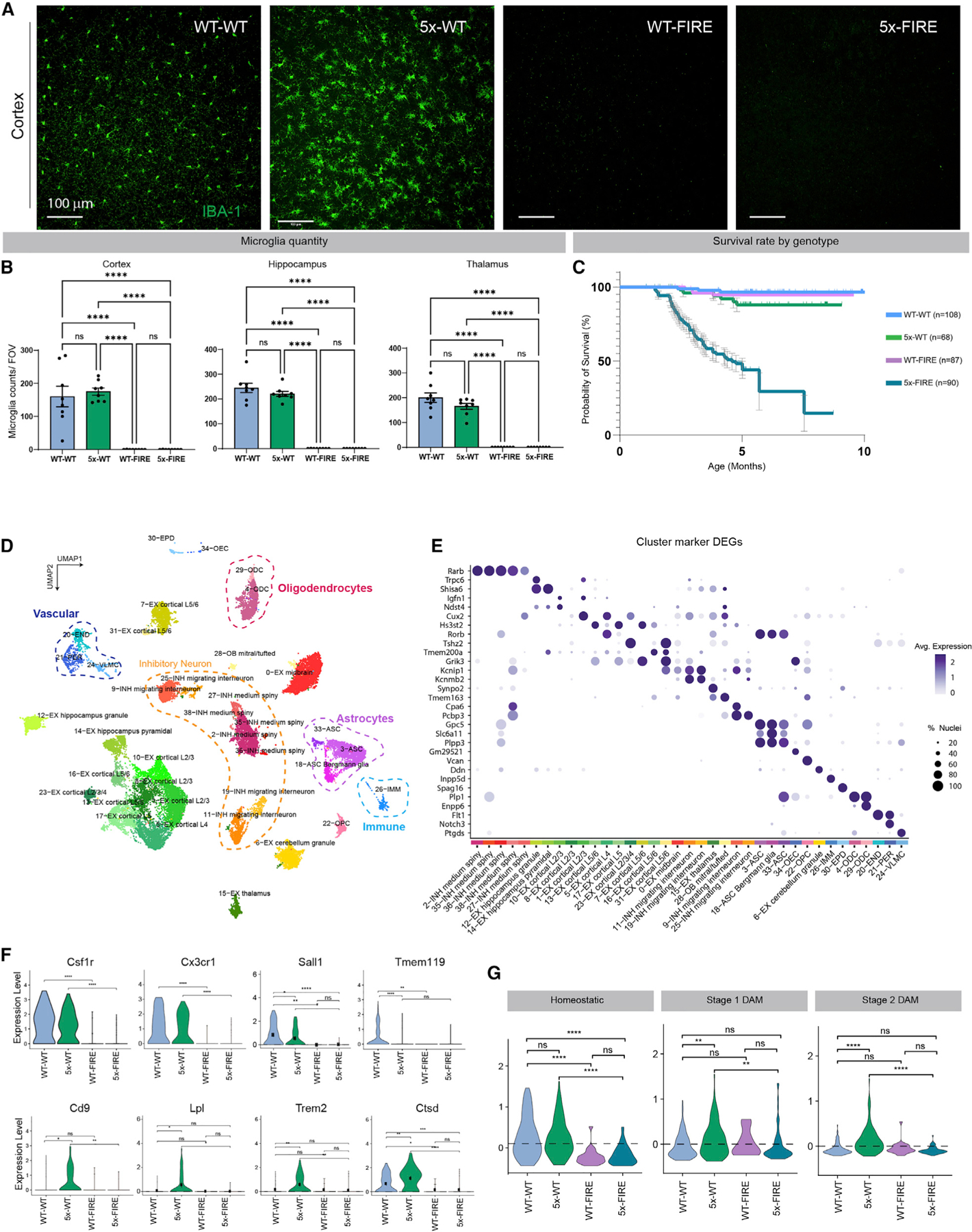
Genetic absence of microglia in AD mice induces premature lethality and alters cell-specific transcriptional states (A) Brains form 5–6-month-old mice (n = 8/group) were stained with IBA-1 (green). Representative confocal images from the cortex demonstrate a homeostatic distribution of IBA-1 immunoreactive microglia in WT-WT mice, a more activated clustering of microglia in 5x-WT mice, and absence of microglia in WT-FIRE and 5x-FIRE mice. (B) FIRE mice lack microglia throughout the brain as quantified within the cortex, hippocampus, and thalamus. (C) Kaplan-Meier survival analysis reveals early lethality in 5x-FIRE mice, with fewer than 29% of mice remaining alive at 6 months of age and only 15% remaining alive at 7.5 months. In contrast, WT-WT, 5x-WT, and WT-FIRE mice exhibit minimal lethality. (D) Uniform manifold approximation and projection (UMAP) of snRNA-seq analysis of 5–6-month-old mice (n = 8/group) provides transcriptomic evidence of 37 distinct clusters, including multiple neuronal subtypes, several astrocyte and oligodendrocyte subtypes, and endothelial and immune cell clusters. (E) A dot plot of the highest expressed gene for each cluster; size of dots indicates percent of cells expressing that gene; color indicates relative expression levels. (F) The absence of microglia in WT-FIRE and 5x-FIRE mice is further confirmed by lack of CSF1R, CX3CR1, SALL1, and TMEM119 gene expression, among others. In addition, increased expression of several disease-associated microglial (DAM) transcripts, including CD9, LPL, TREM2, and CTSD, is observed within the 5x-WT group but not within 5x-FIRE mice. (G) Whereas 5x-WT mice exhibit induction of both stage 1 and 2 DAM module genes, 5x-FIRE show no such induction. Scale bars, 100 µm in (A). All data presented as mean ± SEM. *p ≤ 0.05, **p ≤ 0.01, ***p ≤ 0.001, ****p ≤ 0.0001.

Microglia are reliant on CSF1R signaling for differentiation, proliferation, and survival (Elmore et al., 2014; Hume et al., 2020; Rojo et al., 2019). Constitutive deletion of CSF1R results in an absence of microglia but also leads to postnatal lethality and developmental defects, including hypomyelination and ventricular enlargement (Dai et al., 2002; Patkar et al., 2021). Conditional deletion of CSF1R, inducible diphtheria toxin models, and CSF1R antagonists provide alternative approaches. However, these models induce the rapid death of adult microglia leading to pleotropic effects on astrocytes and promoting T cell recruitment to the brain (Elmore et al., 2014;Marino Lee et al., 2021; Unger et al., 2018). In addition, surviving microglia quickly proliferate to repopulate the microglial niche so that the long-term consequences of microglia absence with such models cannot be effectively studied (Bruttger et al., 2015; Han et al., 2020; Lund et al., 2018; Pons et al., 2020, 2021). As an alternative, we have utilized a recently developed mouse model that harbors a deletion of the FIRE enhancer within the *Csf1r* locus. The resulting FIRE mice lack microglia (Figure 1) but otherwise exhibit normal brain anatomy and health (Rojo et al., 2019).

### AD mice that genetically lack microglia exhibit premature lethality

To produce an AD mouse model that genetically lacks microglia, we crossed FIRE knockout mice with 5xfAD transgenic mice that harbor familial AD mutations in amyloid precursor protein (APP) and presenilin 1 (PSEN1) and develop robust parenchymal amyloid pathology (Oakley et al., 2006). F1 progeny were further crossed to produce mice carrying four key genotypes: wild type for both alleles (WT-WT), 5xFAD hemizygous and wild type for *Csf1r* (5x-WT), wild type for the 5xfAD transgene and homozygous *Csf1r* ^ΔFIRE/ΔFIRE^ (WT-FIRE), and 5xFAD hemizygous and homozygous *Csf1r*^ΔFIRE/ΔFIRE^ (5x-FIRE). To confirm the absence of microglia, cohorts of each genotype were examined by confocal microscopy and flow cytometry. Whereas WT-WT mice exhibit a typical tiled distribution and ramified morphology of IBA-1^+^ microglia, 5x-WT mice exhibit a more activated clustering of microglia as they respond to amyloid plaques (Figure 1A). In WT-FIRE and 5x-FIRE groups, microglia remained absent throughout the brain (Figures 1A and 1B). This finding indicates that Aβ pathology does not induce the recruitment of IBA-1^+^ monocytes or macrophages from the periphery, even in the presence of an empty microglial niche. Flow cytometry analysis further confirmed these results, demonstrating a complete loss of homeostatic (P2RY12^+^/TMEM119^+^) microglia in both WT-FIRE and 5x-FIRE mice (Figure S1A). Most surprisingly, Kaplan-Meier analysis indicated that less than 29% of 5x-FIRE (n = 90) mice remained alive at 6 months of age, whereas WT-WT (n = 108), 5x-WT (n = 68), and WT-FIRE (n = 87) mice exhibited minimal premature mortality (Figure 1C). This striking finding indicates that the absence of microglia per se has no adverse impact on lifespan, but the concurrent presence of Aβ pathology with microglial absence drives premature mortality.

### Single-nucleus RNA sequencing reveals cell-type-specific expression signatures and confirms the absence of microglia in WT-FIRE and 5x-FIRE mice

We next sought to identify transcriptional changes associated with a genetic depletion of microglia in 5xfAD mice.

Therefore, we performed snRNA-seq using split-pool ligation-based transcriptome sequencing (SPLiT-seq) of 5–6-month-old mice (n = 8 per genotype; 4 female [F]/4 male [M]). After rigorous quality control filtering, we retained 30,442 high-quality single-nucleus transcriptomic profiles, which we grouped into 37 biologically relevant cell clusters using dimensionality reduction and Leiden clustering (Figures 1D and S2). Cell clusters were well mixed across genotype and sex, revealing that our analysis grouped cells based on each cell’s underlying gene expression state rather than confounding technical factors (Figure S2). Cell-type identities of each cluster were annotated based on two complementary approaches: an unbiased marker gene analysis (Figure 1E) and a supervised machine learning approach to predict each cell’s identity based on a high-quality annotated reference dataset of the mouse brain (Figure S3) (Rosenberg et al., 2018). Using this approach, we resolved transcriptional profiles of many different neuronal, glial, and vascular cell types, including region-specific excitatory neurons, inhibitory neurons, multiple subtypes of astrocytes and oligodendrocytes, and an immune cell population containing signatures of microglia and other immune cells. For example, marker gene analysis highlighted cluster-specific expression of known markers, such as RAR-related orphan receptor B (*Rorb*) in cortical layer 4 excitatory neurons and retinoic acid receptor β (*Rarb*) in medium spiny neurons (Figure 1E). Several additional transcripts enriched in each cluster are shown in Figure S2I, and all significantly enriched genes for each cluster between the four genotypes are available via Mendeley (https://doi.org/10.5281/zenodo.6565145).

As shown previously (Rojo et al., 2019), and consistent with a complete loss of microglia, FIRE and 5xFIRE mice lacked detectable expression of microglia-expressed transcripts, including *Csf1r*, *Cx3cr1*, *Sall1*, and *Tmem119*, among others (Figure 1F). As expected, *Tmem119* and *Sall1*, two homeostatic microglial genes, were diminished in 5x-WT mice versus WT-WT mice, whereas DAM genes *Cd9*, *Lpl*, *Trem2*, and *Ctsd* were upregulated in 5x-WT microglia. We did not, however, detect reduced expression of *Cx3cr1* and *Csf1r* in 5x-WT mice, despite prior reports that these genes are also reduced in 5x mice (Keren-Shaul et al., 2017). This discrepancy likely results from two technical differences: (1) snRNA-seq analysis of all brain cells likely provides diminished sensitivity in comparison with the previously used CD45^+^ immune cell enrichment approach, and (2) a recent report demonstrates that microglia genes are underrepresented in single-nuclei sequencing versus whole-cell sequencing methods (Thrupp et al., 2020). To further examine the potential induction of DAM genes, we calculated gene module scores for homeostatic, stage 1 DAM, and stage 2 DAM genes. This analysis further revealed a significant induction of both DAM stages in 5x-WT mice versus WT-WT mice and a significant reduction of all three microglial modules in 5x-FIRE mice (Figure 1G). Thus, the great majority of transcriptional changes detected in our snRNA-seq analysis of 5x-WT microglia are highly consistent with prior reports of amyloid-induced changes.

One previous study reported that CSF1R may also be expressed in neurons (Luo et al., 2013), although a recently developed CSF1R-reporter model revealed no neuronal expression (Grabert et al., 2020). To further determine whether the FIRE deletion induces differential expression of CSF1R within neurons, we examined our snRNA-seq dataset. This analysis revealed no detectible expression of CSF1R mRNA in neurons or any other cell type beside the immune cell cluster (data not shown). Thus, it appears CSF1R mRNA expression within the murine brain is restricted to immune cells, and amyloid pathology does not induce non-immune CSF1R expression.

### Flow cytometry further demonstrates the loss of microglia and reveals minimal changes in peripheral immune populations

Because CSF1R is expressed in other myeloid cell populations, it is important to determine whether FIRE mice exhibit alterations in any other immune cells beside microglia. We therefore performed flow cytometry on several tissues collected from each of the four genotypes (Figure S1). Analysis of CD11b^+^/CD45int expression is commonly used to identify microglia but also detects border-associated macrophages (BAMs). As expected, CD11b^+^/CD45int cells were greatly reduced in both FIRE groups, although a few residual cells remained, likely representing meningeal and perivascular macrophages, which were previously shown to be unaffected in FIRE mice (Rojo et al., 2019). Importantly, when all CD11b/CD45 double-positive cells were further examined for expression of the microglia markers P2RY12 and TMEM119, we observed a complete absence of P2RY12^+^/TMEM119^+^ cells in both WT-FIRE and 5x-FIRE groups, further confirming the loss of microglia in FIRE mice (Figure S1A). To determine whether the FIRE enhancer deletion might affect other peripheral immune cells, we also performed flow cytometry on spleen, deep cervical lymph nodes, and bone marrow samples. Each of these analyses revealed no significant differences in CD8 T cells, CD4 T cells, monocytes/macrophages, neutrophils, B cells, and natural killer (NK) cells across all four groups of mice (Figures S1B–S1E). We did, however, detect a small but significant increase in dendritic cell numbers specifically within the spleen of 5x-WT, WT-FIRE, and 5x-FIRE groups in comparison with WT-WT mice. The precise impact of this relatively subtle change remains unclear, but it seems unlikely that this would contribute to the observed brain phenotypes. Taken together, these flow cytometry experiments further confirmed the loss of microglial populations in FIRE mice with little to no detectable impact on other peripheral immune cell types.

### Absence of microglia reduces plaque intensity and insoluble Aβ but promotes the development of cerebral amyloid angiopathy (CAA)

**Figure 2.**
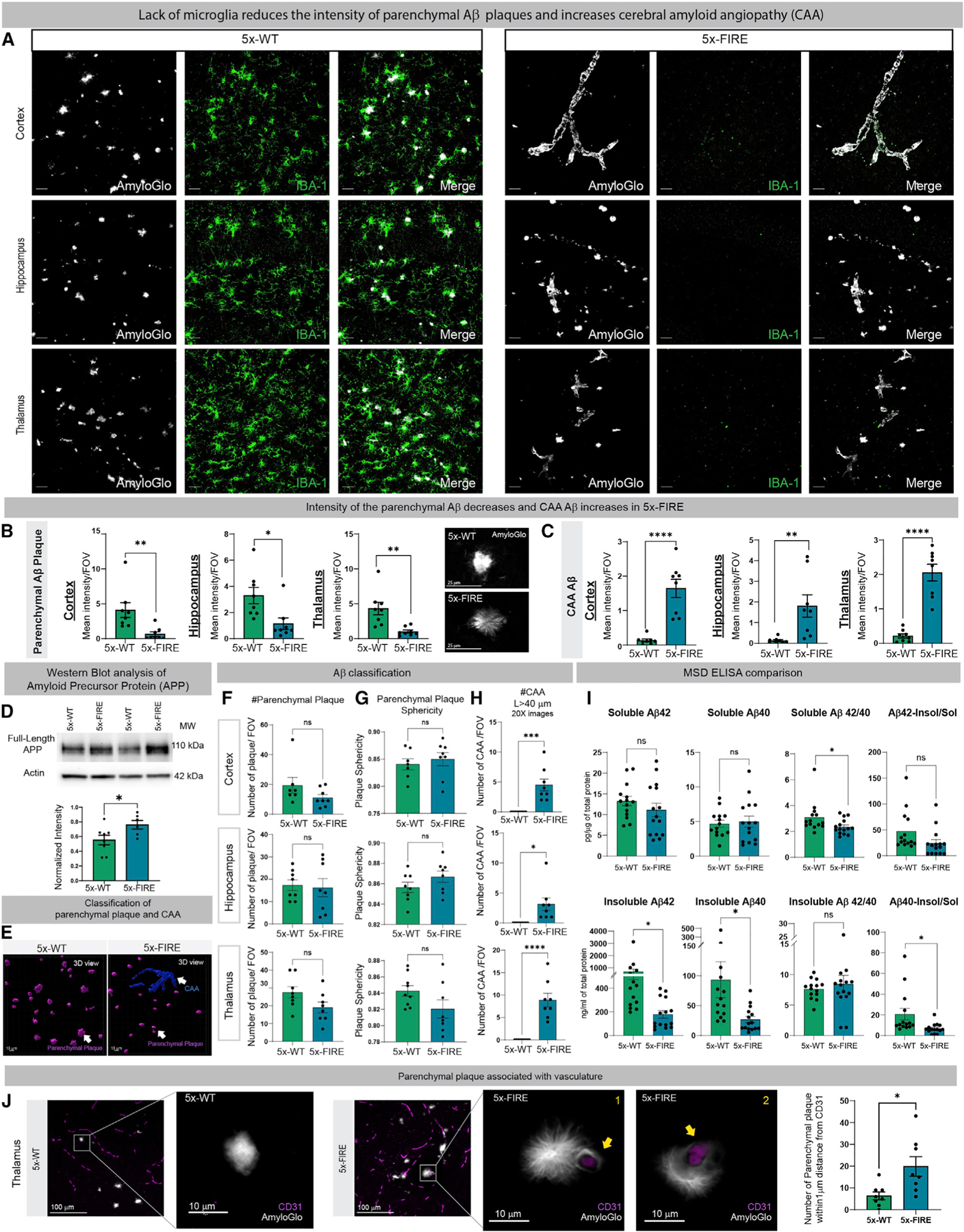
Microglial-deficient AD mice exhibit reduced intensity of parenchymal plaques and diminished insoluble Aβ but a robust induction of cerebral amyloid angiopathy (CAA) (A) The 5–6-month-old 5x-WT mice exhibit parenchymal plaque deposition (Amylo-Glo, white) and clustering of IBA-1 microglia (green). (B and C) In contrast, 5x-FIRE mice exhibit diminished plaque intensity and more diffuse morphology (B) and a robust induction of CAA (C) within all three brain regions examined. (D) Western blots reveal a small but significant increase in human APP protein expression in 5x-FIRE mice. (E–G) (E) Imaris image analysis was used to further classify parenchymal versus vascular amyloid pathology, revealing no significant differences in plaque number (F) or sphericity (G). (H) However, the number of CAA deposits (H) was significantly increased in 5x-FIRE mice. (I) ELISA analysis further reveals significantly reduced levels of insoluble Aβ40 and Aβ42. (J) Whereas 5x-WT plaques are only occasionally observed adjacent to CD31^+^ blood vessels, 5x-FIRE plaques are more frequently associated with blood vessels. Scale bars, 25 µm in (A) and (B); 15 µm in (E); and 100 µm and 10 um in (J). All data presented as mean ± SEM. *p ≤ 0.05, **p ≤ 0.01, ***p ≤ 0.001, ****p ≤ 0.0001.

Amyloid plaques can exhibit varying features, including dense core and diffuse morphologies. Dense core plaques are predominately composed of fibrillar Aβ aggregates and readily detected with ThioS or Amylo-Glo (D’Andrea et al., 2004; DeTure and Dickson, 2019). In contrast, diffuse plaques are typically associated with less compact protofibrils and Aβ oligomers and stain more weakly with ThioS/Amylo-Glo but can be readily detected with Aβ antibodies. To further understand the impact of microglial absence on dense core plaques, Amylo-Glo labeling was performed on 5–6-month-old mice (Figure 2A), revealing a significant reduction of parenchymal plaque intensity in 5x-FIRE mice within the cortex, hippocampus, and thalamus (Figures 2A and 2B). 5x-FIRE plaques also exhibited a more filamentous, diffuse distribution in comparison with the more compact and intense morphology of 5x-WT plaques. These morphological and intensity changes in parenchymal plaques were further accompanied by a marked increase in CAA intensity within the cortex, hippocampus, and thalamus (Figure 2C). To determine whether changes in amyloid intensity might be influenced by altered human APP expression, western blots were performed, revealing a small but significant increase in APP holoprotein expression in 5x-FIRE mice (Figure 2D). Therefore, the observed reduction in Aβ plaque intensity in 5x-FIRE mice cannot be attributed to reduced APP expression.

Next, Imaris image analysis was used to further classify Aβ pathology as parenchymal plaques or CAA (Figures 2E–2H and S4). Despite the reduction in the intensity of parenchymal plaques, no differences in the total number of parenchymal plaques or their sphericity were detected (Figures 2F and 2G). In contrast, amyloid deposits with a length ≥40 µm, indicative of CAA pathology, were increased in all three brain regions of 5xFIRE mice (Figure 2H). The observed histological changes were also accompanied by alterations in biochemical measures of Aβ. Soluble levels of both Aβ40 and Aβ42 detected by ELISA remained unchanged between 5x-WT and 5x-FIRE mice, although a small but significant reduction in the ratio of soluble 42/40 was detected (Figure 2I). However, a significant reduction in both Aβ40 and Aβ42 was detected within the insoluble fractions of 5x-FIRE mice, consistent with the previously observed reduction in parenchymal plaque intensity. Co-labeling for Amylo-Glo and the endothelial cell marker CD31 within the thalamus revealed a significantly increased association between blood vessels and parenchymal plaques in 5x-FIRE mice (Figure 2J). Thus, although total plaque numbers remain unchanged, the development of parenchymal plaques appears to be shifted in 5x-FIRE mice toward a perivascular origin.

### Adult transplantation of wild-type microglia prevents the effects of FIRE deletion on parenchymal Aβ and CAA

**Figure 3.**
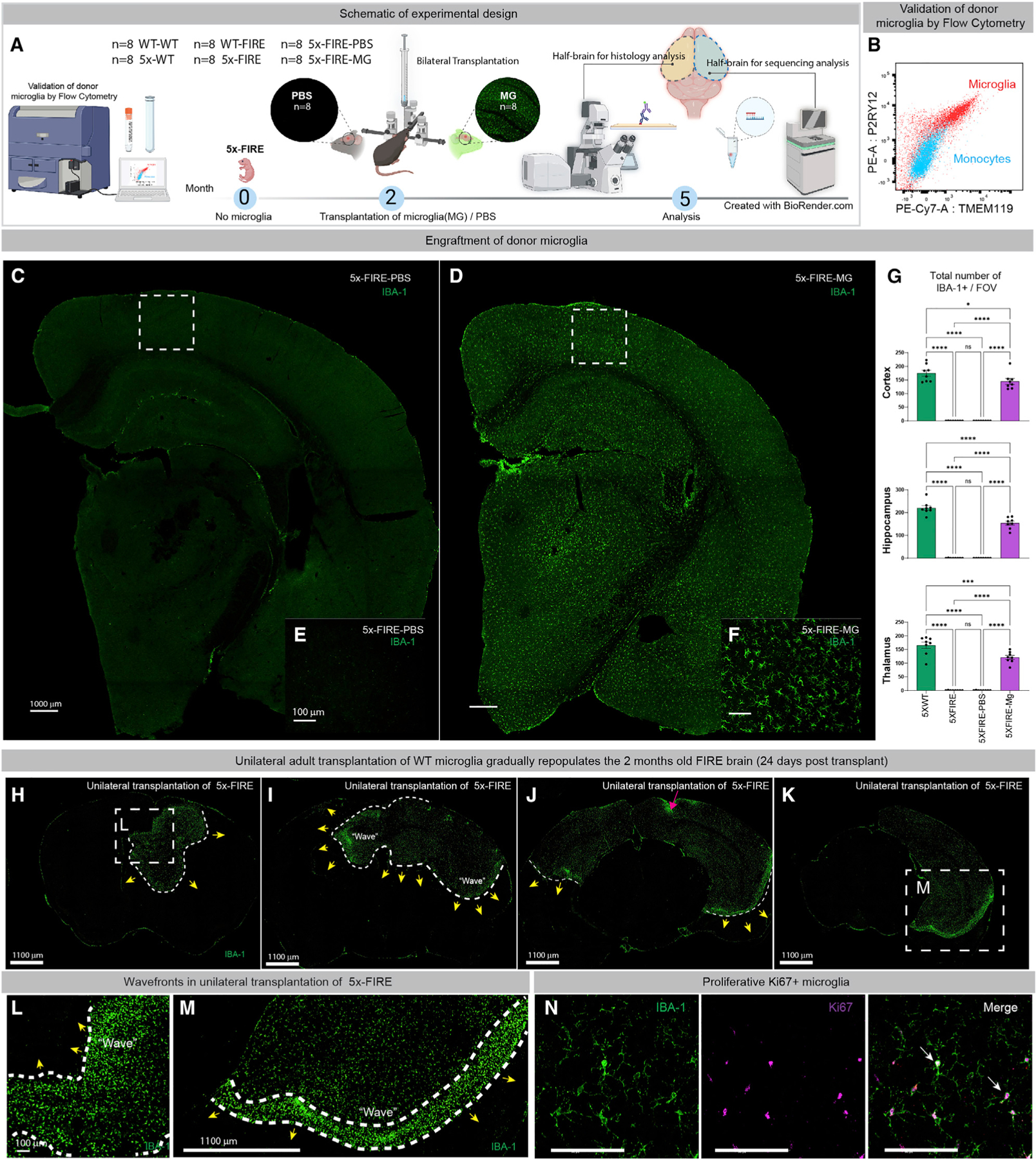
Microglial transplantation leads to brain-wide repopulation of the microglial niche (A) A schematic illustration of the design of adult microglial transplantation studies. (B) Donor wild-type haplotype-matched microglia (red) were examined by flow cytometry in comparison with murine blood-derived monocytes (blue) and found to express high levels of the homeostatic markers P2RY12 and TMEM119. (C–G) (C and E) IBA-1 labeling of 5x-FIRE-PBS mice show very little immunoreactivity. In contrast, 3 months post bilateral transplantation of 160,000 total wild-type microglia, the brains of 5x-FIRE-MG mice are almost fully repopulated, with IBA-1^+^ donor microglia (D and F), quantified in (G). (H–N) Pilot studies were performed to examine the repopulation kinetics 24 days after unilateral transplantation of 80,000 microglia. The red arrowhead in (J) marks the injection site from which microglia migrate and expand into the unoccupied niche. (L and M) Higher-power views of the boxed regions in H and M show a dense wavefront of microglia. (N) Immunolabeling with the mitotic marker Ki67 shows increased levels of proliferation within these wavefront microglia. Scale bars, 1000 µm in (C) and (D), 100 µm in (E) and (F), 1100 µm in (H–K) and (M), and 100 µm in (L) and (N). All data presented as mean ± SEM. *p ≤ 0.05, **p ≤ 0.01, ***p ≤ 0.001, ****p ≤ 0.0001.

Congenital absence of microglia might potentially lead to developmental changes that predispose to amyloid pathology. To exclude this possibility, we examined the impact of adult transplantation of WT murine microglia into 5x-FIRE mice (Figure 3A). Donor microglia derived from postnatal WT C57BL7 mice were characterized by flow cytometry, revealing robust expression of the homeostatic microglia markers P2RY12 and TMEM119 (Figure 3B). At 2 months of age, 5x-WT mice are just beginning to develop parenchymal plaque pathology (Oakley et al., 2006). Therefore, 2-month-old 5x-FIRE mice were randomly assigned to microglia transplantation (5x-FIRE-MG) or PBS control (5x-FIRE-PBS) groups, and 80,000 WT microglia or equivalent volume of PBS vehicle were delivered stereotactically into the hippocampus and overlying cortex of each hemisphere. Three months later, 5-month-old mice were sacrificed (n = 8/group, 4F/4M), and confocal analysis of IBA-1 immunoreactivity performed. Remarkably, bilateral injection of donor microglia led to widespread and near complete engraftment of the previously empty microglial niche (Figures 3C–3G). Pilot, shorter-term, 24-day engraftment studies were also performed to better understand microglial repopulation kinetics. Following unilateral transplantation, we observed partial microglial repopulation emanating from the injection site and the appearance of ‘‘wave-fronts’’ of proliferative Ki67^+^ microglia adjacent to unoccupied regions of the brain (Figures 3H–3N). These wavefronts appear to be highly similar to those recently described during microglial repopulation following complete pharmacological depletion (Hohsfield et al., 2021). Thus, WT microglia appear capable of repopulating the entire brain of FIRE mice within 3 months.

**Figure 4.**
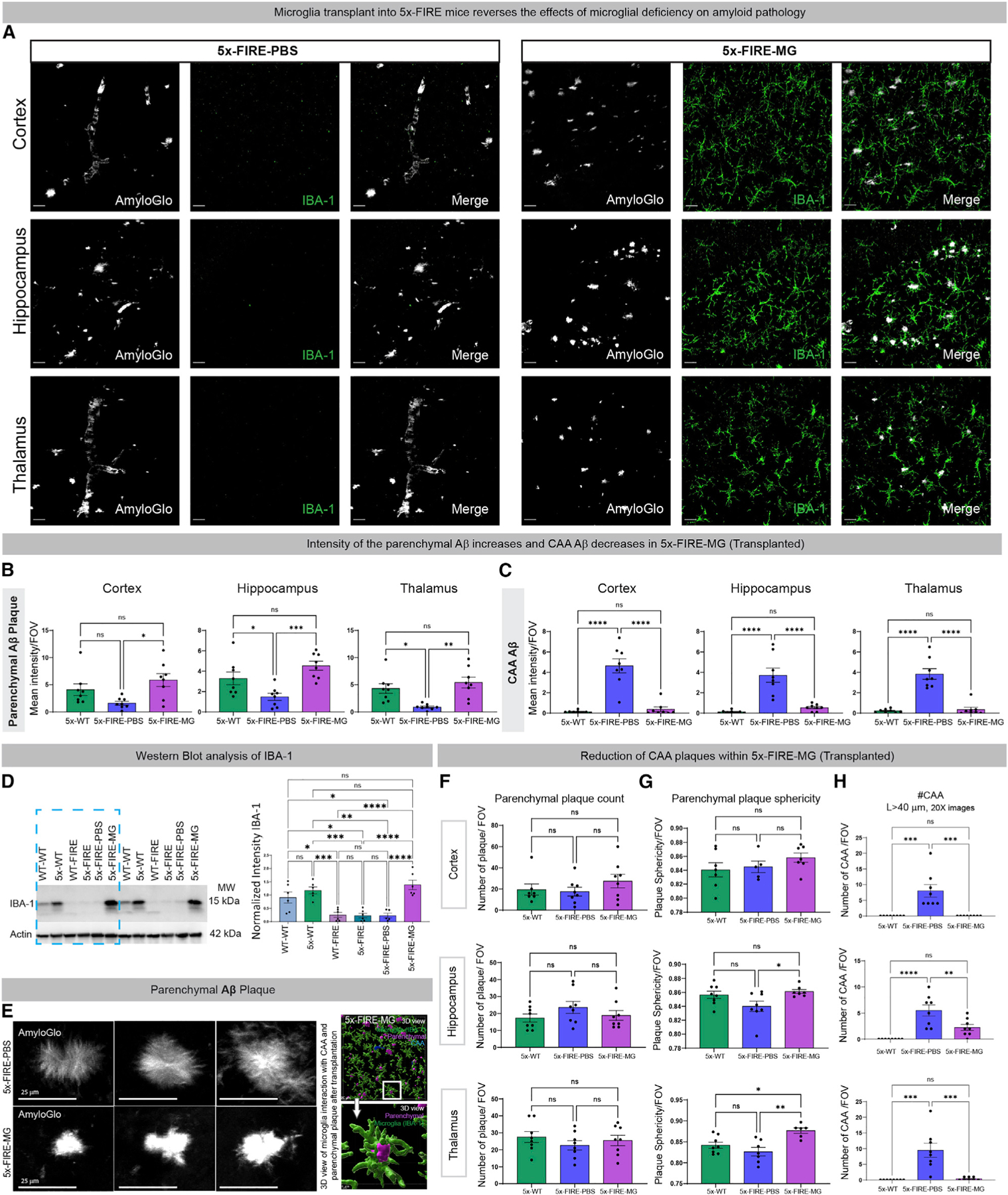
Adult transplantation of wild-type donor microglia prevents the effects of microglial deficiency on amyloid pathology (A–C) (A) Microglial repopulation leads to a reversal of the previously observed changes in amyloid distribution, increasing parenchymal Aβ intensity back to 5X-WT levels (B), while concurrently decreasing CAA (C) in comparison with PBS-injected control 5x-FIRE mice. (D) Western blot analysis of IBA-1 further demonstrates the loss of microglia in FIRE mice and the return of IBA-1 signal following microglial transplantation. (E) Representative high-power images of parenchymal plaques further demonstrated a shift in morphology from more diffuse filamentous plaques in 5x-FIRE-PBS mice toward more compact, intense morphology in 5x-FIRE-MG mice. (F) Transplantation of microglia has no effect on total plaque numbers. (G and H) In contrast, plaque sphericity within both the hippocampus and thalamus was enhanced by microglial transplantation (G) and the number of CAA deposits in all three brain regions was significantly reduced (H). Scale bars, 25 µm in (C); 25 µm, 20 µm, and 4 µm in (G). All data presented as mean ± SEM. *p ≤ 0.05, **p ≤ 0.01, ***p ≤ 0.001, ****p ≤ 0.0001.

Importantly, we also found that delivery of donor microglia entirely reversed the changes in parenchymal versus CAA pathologies and diffuse plaque morphology within the cortex, hippocampus, and thalamus (Figures 4A–4C). To further confirm that FIRE mice exhibit a brain-wide loss of microglia, and that microglial transplantation can reverse that loss, western blotting for IBA-1 was performed. As expected, IBA-1 levels were significantly reduced in FIRE mice and FIRE mice that received PBS injections but restored to levels equivalent to 5x-WT mice following microglia transplantation (Figure 4D). As a result, 5x-FIRE-MG mice also exhibited more compact plaque morphology and a recapitulation of the close juxtaposition between parenchymal plaques and microglia (Figure 4E). Just as the absence of microglia had no effect on total plaque numbers, microglial transplantation also had no impact on this measure (Figures 4F, S4E, and S4I). However, adult replacement of microglia did have a significant impact on the sphericity of plaques in both the hippocampus and thalamus (Figures 4G and S4L), supporting the concept that microglia contribute to the compaction of parenchymal plaques. Because Amylo-Glo labels dense core plaques more robustly than diffuse plaques, we also examined total amyloid pathology via immunofluorescent staining with the human-specific antibody 82 × 10^1^. This analysis revealed highly similar results to our Amylo-Glo analysis, including no significant changes in plaque number between any 5x group but a robust reduction in CAA via either length- or area-based analyses (Figure S5).

### Transplanted microglia exhibit altered morphology and increased association with blood vessels

**Figure 5.**
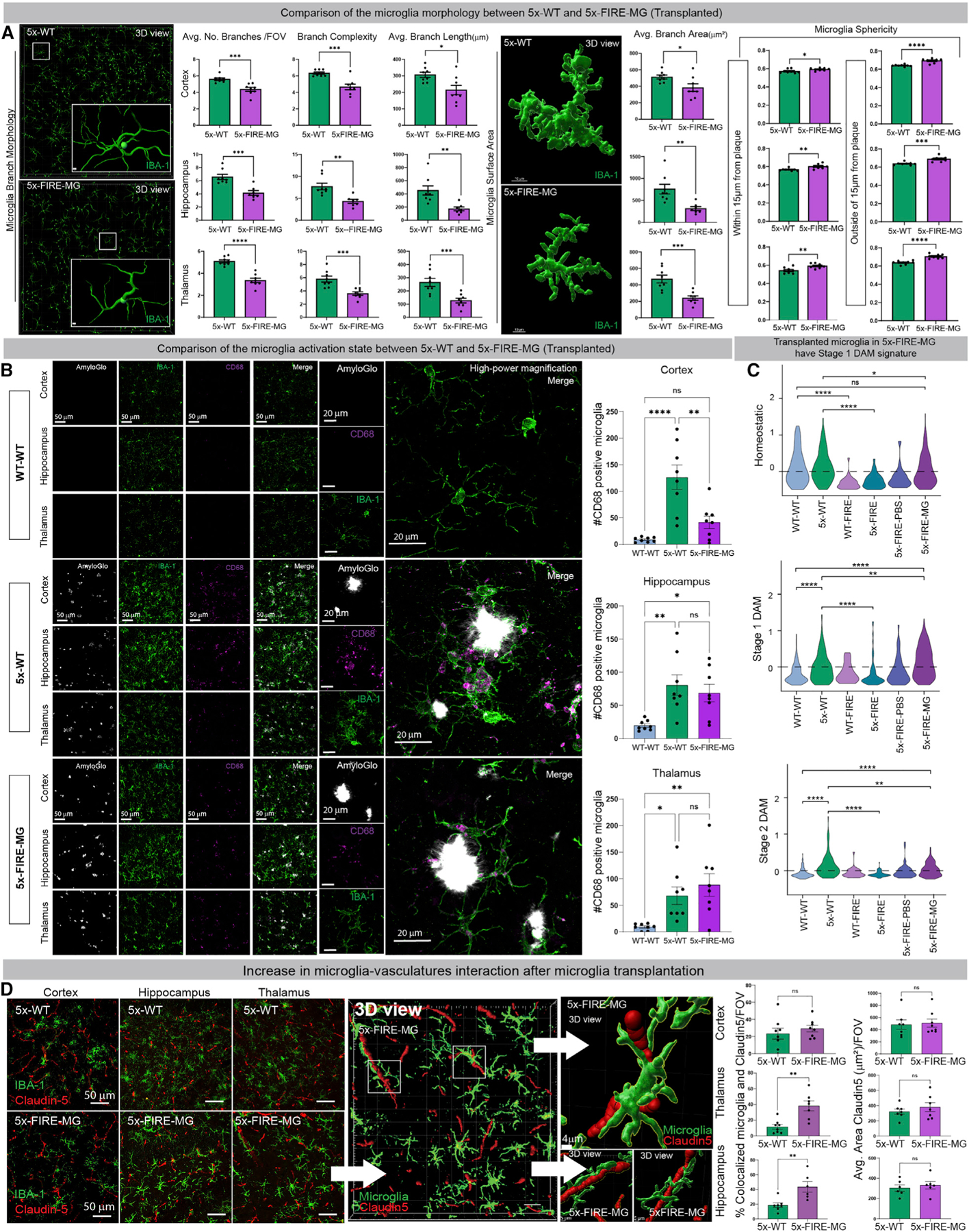
Transplanted microglia exhibit differential morphology, altered CD68 expression, and increased association with blood vessels (A) Imaris analysis of microglial branch number, complexity, length, and area reveals reductions in each of these measures in transplanted microglia versus endogenous 5x-WT microglia. Transplanted microglia also exhibit reduced branch complexity and increase sphericity, both adjacent to (≤15 µm) and more distant from (>15 µm) Aβ plaques. (B) Immunolabeling for CD68 revealed a significant reduction in transplanted microglia within the cortex, but no changes within the hippocampus or thalamus. (C) snRNA-seq demonstrates that transplanted microglia adopt both a DAM1 and DAM2 transcriptomic response in 5x-FIRE-MG mice. (D) Examination of Claudin-5 and IBA-1 immunoreactivity reveal an increased association between transplanted microglia and blood vessel endothelial cells within the thalamus and hippocampus (thalamus shown in Imaris 3D view) but no change in Claudin-5 immunoreactive blood vessel area. Scale bars, 1 µm and 10 µm in (A), 50 µm and 20 µm in (B), and 50 µm and 4 µm in (D). Graphical data presented as mean ± SEM.*p ≤ 0.05, **p ≤ 0.01, ***p ≤ 0.001.

Transplanted microglia did not entirely recapitulate the morphology of endogenous 5x-WT microglia as they exhibited decreased numbers of branches, reduced branch complexity, shorter branch length, and reduced branch area (Figure 5A). The sphericity of transplanted microglia was also increased regardless of whether they were proximal or distal to parenchymal plaques. To further understand whether transplanted microglia also exhibit differential phagocytic activity, we examined levels of the microglial phagolysosomal protein CD68, revealing a significant decrease in CD68 within the cortex of 5x-FIRE-MG mice but no differences within the hippocampus and thalamus (Figure 5B).

To identify gene expression changes brought on by microglial transplantation, we performed a combined snRNA-seq analysis of microglia and PBS-transplanted groups with the original four genotypes. Following a similar analytical workflow, we revealed 43 cell clusters and an overall highly similar cell-type composition to our previous analysis (Figure S6). Interestingly, this further analysis revealed significantly increased DAM1 and DAM2 gene signatures in transplanted microglia versus endogenous 5x-WT microglia (Figure 5C). Within the hippocampus and thalamus, transplanted microglia were also closely associated with Claudin-5^+^ vasculature (Figure 5D). Collectively, these data suggest that transplanted microglia adopt a differential activation state from endogenous 5x-WT microglia, likely in response to CAA pathology.

### Meningeal and perivascular macrophage populations remain unchanged in FIRE mice

Meningeal and perivascular macrophages were previously reported to be unchanged in FIRE mice (Rojo et al., 2019). However, the addition of amyloid pathology might further impact these BAM populations. We therefore quantified meningeal and perivascular macrophage numbers by examining CD206 (Mrc1). This analysis clearly demonstrated the sensitivity of CD206 for meningeal and perivascular macrophages but also revealed no examples of CD206^+^ cells within the parenchyma that were not immediately adjacent to CD31^+^ blood vessels (Figure S7). As CD206 also labels brain-infiltrating macrophages, this finding further supports the notion that little to no macrophage infiltration occurs in 5xfAD mice even when microglia are absent. Importantly, quantification of total CD206 numbers across all six groups of mice also revealed no significant differences in meningeal and perivascular macrophage density (Figure S7). Thus, the effects of the FIRE deletion on amyloid pathology do not involve alterations in meningeal or perivascular macrophage density.

### Few changes in astrocyte numbers are detected in 5x-FIRE mice

Astrocytes can also respond to amyloid plaques and microglial-astrocytic crosstalk plays an important role in AD pathogenesis (Liddelow et al., 2017; Sadick et al., 2022). We therefore examined the density of glial fibrillary acidic protein (GFAP) immunoreactive astrocytes within the cortex, hippocampus, and thalamus and quantified astrocytic proximity to amyloid plaques (Figure S8). As expected, the accumulation of amyloid pathology in 5x-WT mice increased the density of GFAP^+^ astrocytes within the cortex and thalamus in comparison with WT-WT mice. However, no differences in astrocytes density were detected between 5x-WT and 5x-FIRE mice within the cortex and hippocampus. In contrast, astrocyte numbers within the thalamus were significantly reduced in 5x-FIRE versus 5x-WT mice and partially restored following microglial transplantation (Figure S8A). To further determine whether absence of microglia impacts the association of astrocytes with Aβ pathology, we examined the proximity of GFAP^+^ astrocytes to Aβ plaques but detected no differences within any of the three brain regions examined. Although limited in scope, these data suggest that absence of microglia only subtly impacts the astrocytic response to Aβ pathology in 5x-FIRE mice.

### Co-expression network analysis further implicates vascular dysfunction in 5x-FIRE mice

**Figure 6.**
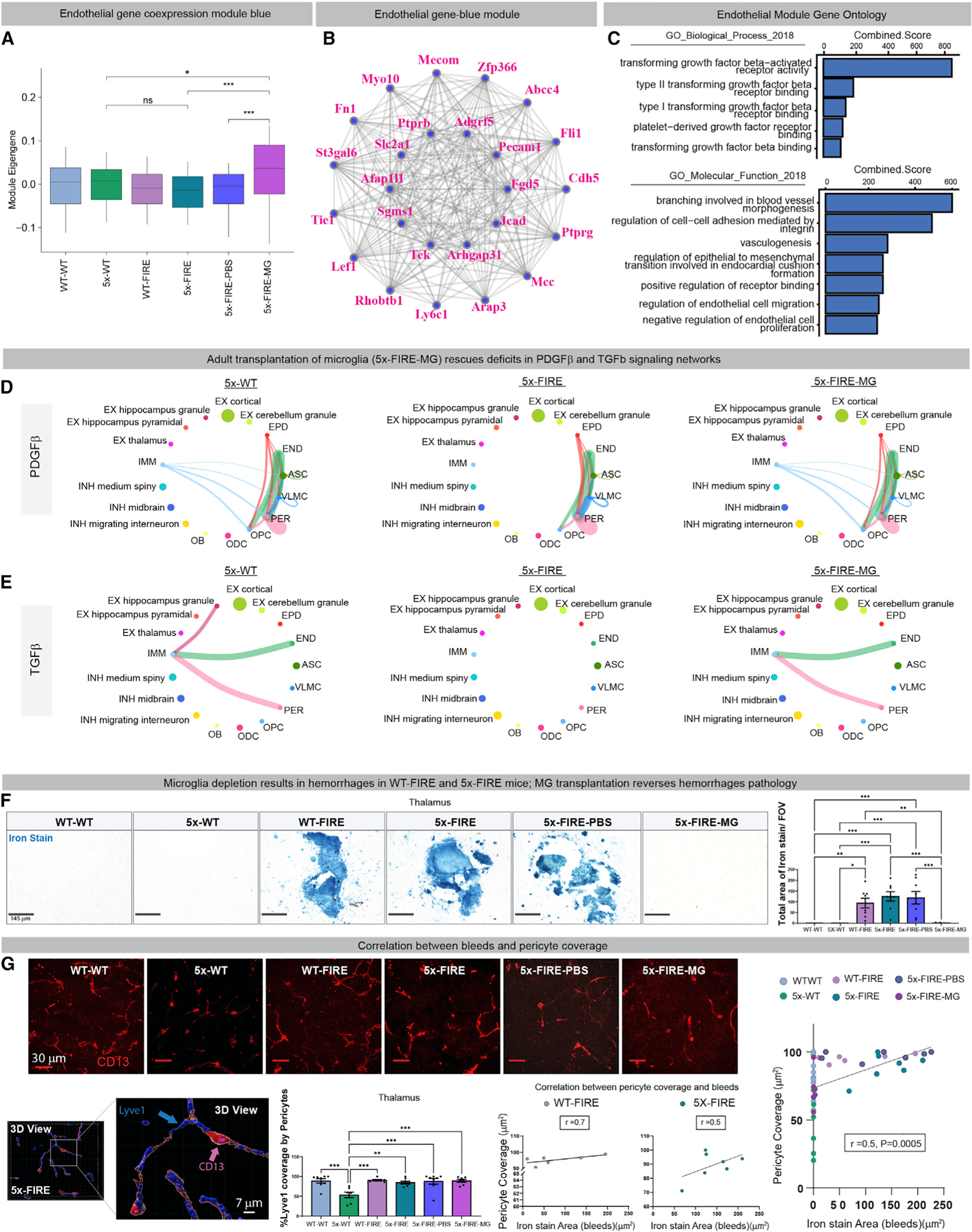
Microglial deficiency induces changes in endothelial gene expression and promotes brain hemorrhages, which can be prevented by adult microglial transplantation (A and B) Co-expression network analysis of snRNA-seq data from 5–6-month-old mice (n = 8/group) reveals significant changes in endothelial cell gene expression between 5x-FIRE mice transplanted with microglia versus PBS-injected 5x-FIRE controls, untranslated 5x-FIRE mice, and 5x-WT mice. (C) Gene ontology analysis further highlights several important pathways that are altered in endothelial cells, including PDGF-β and TGF-β receptor signaling, blood vessel branching, and regulation of endothelial cell proliferation and migration. (D) CellChat analysis was utilized to identify signaling networks between differing cell types, revealing PDGFβ-related connectivity between the IMM microglia and pericyte clusters that was abolished in 5x-FIRE mice but fully restored with microglial transplantation. (E) Analysis of TGF-β signaling likewise exhibited a strong connectivity with both pericytes and endothelial cells that was lost in 5x-FIRE mice but restored by transplantation. (F) Prussian blue iron staining revealed clear evidence of hemorrhages in WT-FIRE, 5x-FIRE, and 5x-FIRE-PBS mice that was completely prevented by adult microglial transplantation. (G) Given our CellChat identification of pericytes as microglial signaling recipients, pericyte coverage of endothelial cells was examined via CD13 labeling (red) of pericytes adjacent to Lyve1^+^ blood vessels (blue). This analysis confirmed the previously reported reduction in AD transgenic mice pericytes but revealed no differences between WT-WT and FIRE groups. However, regression analysis comparing pericyte coverage to Prussian blue^+^ hemorrhages revealed a positive correlation between these measures. Scale bars, 145 µm in F, 30 µm in (G), and 7 µm in the 3D view in (G). Data in (A), (F), and (G) presented as mean ± SEM.*p ≤ 0.05, **p ≤ 0.01, ***p ≤ 0.001.

Next, we sought to further characterize systems-level changes in the gene expression landscape between all six groups of mice and within specific cell populations. We therefore performed weighted gene co-expression network analysis (WGCNA), taking measures to account for the sparsity of snRNA-seq measurements by constructing aggregate metacells in each major cell type (STAR Methods). We identified co-expressed gene modules in excitatory neurons, inhibitory neurons, astrocytes, oligodendrocytes, and vascular cells and we revealed significant changes among the different genotypes (Figures S9–S13). Given the marked effects of FIRE deletion on CAA and the association of transplanted microglia with brain vasculature, we focused our attention on endothelial cells (ENDs). Examination of one module, in particular (END-M1, blue module, Figure S9), revealed a significant increase in the module eigengene in 5x-FIRE-MG compared with 5x-FIRE groups (Figure 6A). This module contained many important blood-vessel-associated genes, including *Tie1*, *Cdh5*, and *Lef1*, and was specifically expressed in the brain vasculature cell compartment (Figure 6B). Gene ontology analysis of this module pointed toward significant changes in transforming growth factor beta (TGF-β) and platelet-derived growth factor beta (PDGF-β) pathways (Figure 6C), both of which have been strongly implicated in neurovascular function (Arnold et al., 2014; Buckwalter et al., 2002; Kandasamy et al., 2020; Lindahl et al., 1997). In addition, prior studies have shown that microglia express the highest mRNA levels of these two growth factors within both mouse and human brains (Zhang et al., 2014, 2016). The potential interactions between microglia and vascular cells were further examined using CellChat to quantitatively examine intercellular communication (Jin et al., 2021) (STAR Methods). This analysis revealed a striking loss of PDGF-β-dependent pathways that interconnect microglia (IMM) with pericytes (PERs), vascular leptomeningeal cells (VLMCs), astrocytes (ASCs), and oligodendrocyte precursors (OPCs) within 5x-FIRE mice (Figure 6D). The loss of this PDGF-β signaling pathways was in turn reversed by adult microglial transplantation. Likewise, TGF-β signaling between microglia, PERs, and ENDs was similarly lost in 5x-FIRE mice but restored by microglial transplantation (Figure 6E).

Importantly, our co-expression analysis further revealed transcriptional systems in other cell types that were significantly altered in the 5x-FIRE mice but recovered with microglia transplantation. For example, one module in excitatory neurons (EX-M7, Figure S10), included glutamatergic synapse ontology terms and network hub genes, such as *Satb2* and *Gria4*. Furthermore, an oligodendrocyte progenitor module was significantly altered in the FIRE conditions but unchanged from 5x-WT in the transplanted condition (ODC-M3, Figure S11). ODC-M3 was enriched for GO terms related to amino acid transport, Notch signaling, and extracellular matrix organization, and this module had network hub genes, including *Tnr*, *Atp1a2*, and *Dpp6*. Moreover, several co-expression modules were not significantly altered across genotypes, including myelination-related module ODC-M1. In addition to WGCNA analysis, we performed differential gene expression analysis to compare expression signatures between different genotypes within major cell types (data available via Mendeley https://doi.org/10.5281/zenodo.6565145).

### Absence of microglia promotes intracerebral hemorrhages in 5–6-month-old 5x-FIRE mice

CAA is associated with increased brain hemorrhages in both humans and mice (Jäkel et al., 2017; Zhang-Nunes et al., 2006), and WGCNA analysis further implicated END dysfunction. We therefore performed Prussian blue staining to measure blood-brain barrier integrity. At 2 months of age, WT-WT, 5x-WT, WT-FIRE, and 5x-FIRE show no evidence of hemorrhages (Figures S14A and S14C). Likewise, at 5–6 months of age WT-WT, 5x-WT, and 5x-FIRE-MG groups show no signs of intracerebral hemorrhage (Figure 6F). In contrast, both small and larger hemorrhages were detected in multiple brain regions, in particular the thalamus, in WT-FIRE, 5x-FIRE, and 5x-FIRE-PBS groups (Figure 6F). Given that our cell-signaling analysis showed altered PDGF-β signaling, and the well-established importance of this growth factor in PER survival and function (Lindahl et al., 1997), we next sought to examine PER density. As expected and consistent with prior reports (Sagare et al., 2013), increased vascular leakage was associated with reduced CD13 immunoreactive PER coverage of LYVE1^+^ blood vessels in both the hippocampus and thalamus of 5x-WT mice (Figure 6G). Yet, no changes in PER coverage were detected between WT-WT mice and any of the other 4 groups of mice. However, PER coverage exhibited a positive correlated with Prussian blue staining in both WT-FIRE and 5x-FIRE groups (Figure 6G). Given these findings, we speculate that increased PER coverage in FIRE mice may be associated with a compensatory attempt to respond to other causes of blood-brain barrier (BBB) dysfunction, such as the loss of microglial/endothelial interactions observed in our CellChat analysis (Figures 6D and 6E).

### 5x-FIRE mice develop brain calcification that is prevented by adult microglia transplantation

**Figure 7.**
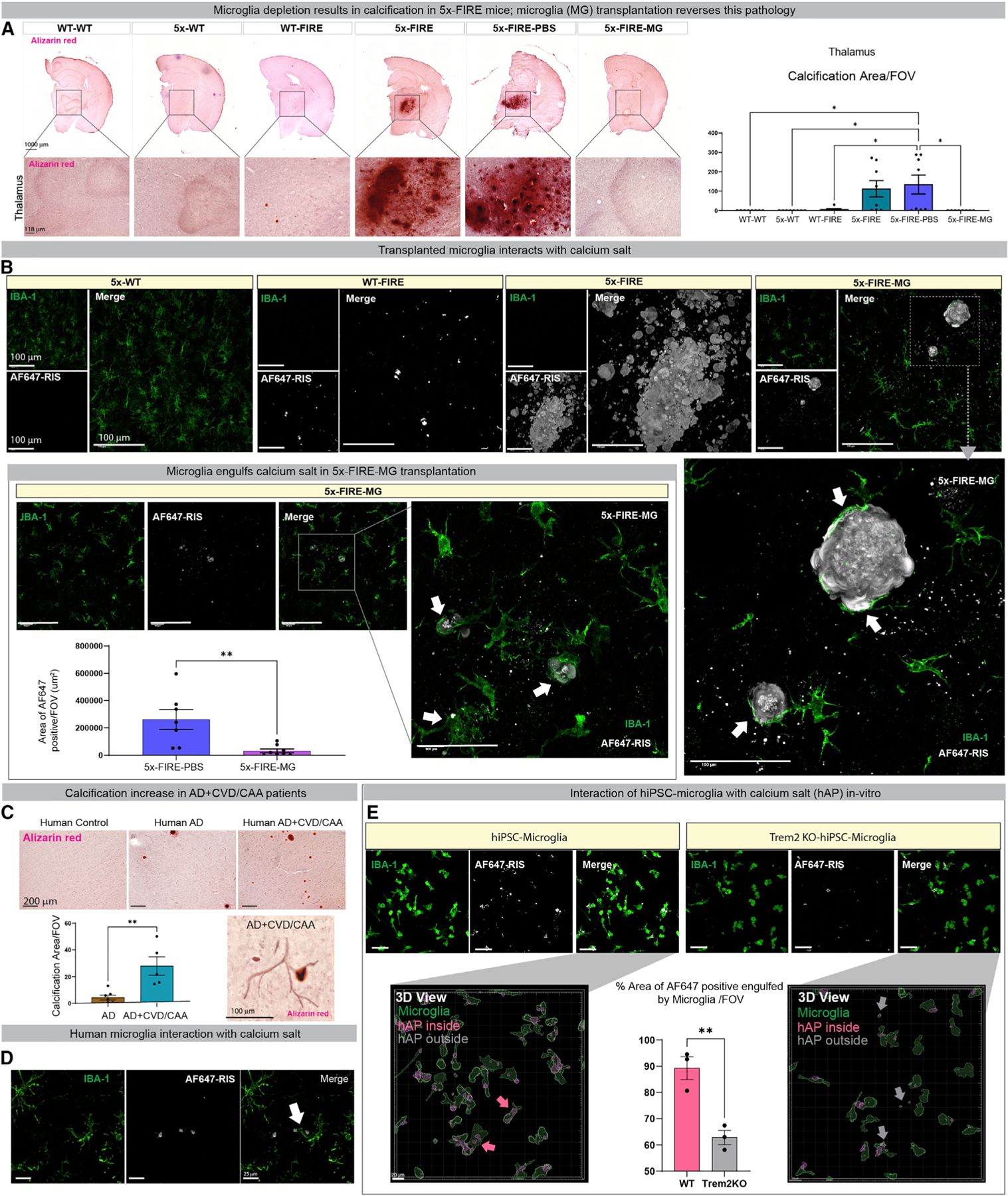
Brain calcification occurs in 5x-FIRE mice and a subset of human AD subjects, and loss of TREM2 impairs microglial phagocytosis of calcium crystals (A) To determine whether other brain vascular pathologies might impact cerebral hemorrhages, we next examined calcium accumulation with alizarin red, revealing a striking increase in calcification within the thalamus of 5x-FIRE mice and 5x-FIRE-PBS mice that was fully resolved in microglial-transplanted 5x-FIRE mice. (B) Excessive calcium accumulates in the form of hydroxyapatite calcium phosphate crystals, which can be detected with the fluorescent indicator AF657-VIS. Confocal imaging of AF657-VIS (white) revealed a complete lack of calcium accumulation in 5x-WT mice, a small increase in hydroxyapatite in WT-FIRE mice, and a substantial increase in 5x-FIRE mice. In contrast, microglial transplanted FIRE-5x mice exhibited greatly decreased hydroxyapatite labeling with remaining calcium crystals (white) often surrounded by IBA-1^+^ microglia (green, arrows) and evidence of microglial phagocytic engulfment of hydroxyapatite observed. (C) To determine the potential implications of this finding for human AD, we examined the relationship between brain calcium accumulation and Aβ plaque pathology in AD cases. (D) This analysis revealed a significant increase in brain calcification within AD patients that exhibit vascular pathologies, with alizarin red calcification often observed adjacent to blood vessels, and microglia (D, IBA-1, green) observed adjacent to AF657-VIS^+^ calcification (white). (E) Lastly, to determine whether microglial expression of TREM2 might influence calcification, WT and *TREM2* knockout iPSC-microglia (GFP, green) were exposed to hydroxyapatite calcium phosphate crystals (HAp, AF647-RIS, gray) and percent internalization determined, revealing a failure of *Trem2* KO microglia to efficiently phagocytose calcium crystals. Scale bars, 1000 µm and 118 µm in (A), 100 µm in (B), 200 µm and 100 µm in (C), 25 µm in (D), and 100 µm and 20 µm in (E), as labeled within the images. All graphs presented as mean ± SEM. *p ≤ 0.05, **p ≤ 0.01

Mutations in either PDGF-β or its receptor PDGF-R-β lead to primary familial brain calcification (PFBC), a neurodegenerative disease that involves accumulation of hydroxyapatite (HAp) calcium crystals within the basal ganglia and thalamus leading to parkinsonian-like symptoms (Tadic et al., 2015). Interestingly, a recent study found that depletion of microglia with a CSF1R antagonist or deletion of the AD microglia risk gene *Trem2* in a PFBC mouse model led to increased brain calcification (Zarb et al., 2021). Thus, microglia likely play an important role in regulating levels of calcium crystals within the brain. Calcification is also a well-established pathological feature of microglial deficiency associated with human recessive *CSF1R* mutations (Guo et al., 2019). At 2 months of age, WT-FIRE and 5x-FIRE mice exhibit no evidence of calcium accumulation (Figures S14B and S14C). Likewise, at 5–6 months of age, WT-FIRE mice continue to show very little alizarin red reactivity (Figure 7A). In contrast, 5x-FIRE mice exhibit a marked increase in calcium accumulation that also persisted in PBS-injected 5x-FIRE mice but was fully reversed in microglia-transplanted 5x-FIRE mice (Figure 7A). Taken together, these data demonstrate that a combined loss of microglia along with amyloid pathology markedly increases brain calcification, which is fully prevented by adult microglial transplantation.

The relationship between microglia and brain calcium accumulation *in vivo* was further examined using Alexa Fluor 647-labeled risedronate (AF647-RIS), a fluorescently modified derivative of bisphosphonate, which binds to HAp calcium crystals (Roelofs et al., 2010). This sensitive approach revealed a small increase in calcium accumulation, particularly within the thalamus of WT-FIRE mice, that was greatly exacerbated in 5x-FIRE mice (Figure 7B). Importantly, and consistent with our alizarin red results, the levels of HAp calcium crystals were significantly reduced in 5x-FIRE mice that had received microglial transplantation versus 5x-FIRE-PBS mice. Interestingly, the few remaining AF647-RIS-labeled deposits detected in 5x-FIRE-MG mice were often surrounded by microglia which could also be observed engulfing HAp calcium crystals (Figure 7B, arrows).

### Calcification is increased in AD subjects with vascular pathologies

Accumulation of calcium deposits in human brains has been described in AD, vascular dementia, and adult-onset leukodystrophies (Ayrignac et al., 2017; Fujita et al., 2003; Sellal et al., 2017). To link the current findings in our mouse model to human clinical pathology, we performed alizarin red staining of 10 subjects that had been diagnosed with AD by the UC Irvine Alzheimer’s Disease Research Center (ADRC). Half of these subjects had known vascular pathologies, including CAA or cardiovascular disease (CVD). Optical density measurements of calcification were then examined within the prefrontal cortex, revealing a significant increase in brain calcification specifically within AD patients that exhibited vascular pathologies (Figures 7C and 7D). Thus, calcification appears to be particularly common in AD patients that co-exhibit vascular disease.

### Deletion of the AD risk gene *TREM2* impairs microglial phagocytosis of calcium crystals

Given recent evidence that *TREM2* can modulate brain calcification (Zarb et al., 2021) and is entirely lost from microglia-deficient mice (Figure 1F), we tested the impact of *TREM2* deletion on the ability of human microglia to respond to and clear pathological calcium deposits. Using a CRISPR-modified isogenic pair of *TREM2*-knockout-induced pluripotent stem cells (iPSCs) (McQuade et al., 2020), we examined the response of human iPSC-microglia to fluorescently labeled HAp calcium crystals. Deletion of *TREM2* led to a significant impairment in the robust calcium crystal internalization seen in control cells (Figure 7E). These exciting data suggest that, just as *TREM2* deletion and mutations impair the ability of microglia to sense, internalize, and degrade Aβ and lipids, so too can this mutation directly impair the clearance of brain calcification. Thus, *TREM2* and perhaps other AD risk genes likely modulate the development of diverse AD-associated pathologies.

## DISCUSSION

Recent studies have carefully examined the impact of AD-associated microglial risk genes on Aβ plaque and neurofibrillary tangle pathologies (Lee et al., 2021; Leyns et al., 2017; Parhizkar et al., 2019, 2019, 2019). However, the influence of microglia on several other AD-associated neuropathologies remains understudied. Here we utilized a genetic model of microglial depletion to examine this important topic, finding compelling evidence that microglia are critically important for prolonging lifespan in AD transgenic mice by limiting the development of CAA, brain calcification, and cerebral hemorrhages. Importantly, the ability of adult microglial transplantation to prevent these pathologies further demonstrates the progressive nature of this disease and suggests that therapeutics designed to modify microglial activity could have important effects on multiple aspects of AD neuropathology and perhaps even lifespan. By examining human AD tissue and iPSC-microglia, we further demonstrate the translational implications of this research, revealing that human microglia likely perform an important protective function in preventing the accumulation of brain calcification and that an AD-associated gene impacts this role.

Many prior studies have utilized CSF1R antagonists, such as PLX5622, to ablate microglia. Somewhat surprisingly, microglial ablation or reduction in AD mouse models has often been shown to confer either minimal or beneficial effects (Bennett et al., 2018; Dagher et al., 2015; Olmos-Alonso et al., 2016; Shi et al., 2019; Spangenberg et al., 2016). However, one recent study showed that ablation of microglia at a young age (1.5–2 months) greatly reduced parenchymal Aβ plaque load but concurrently shifts amyloid distribution toward the vasculature (Spangenberg et al., 2019). Our current data in 5x-FIRE mice recapitulate one key aspect of these findings: a robust increase in CAA. However, PLX5622 treatment also markedly reduced parenchymal plaque load whereas Aβ plaque numbers remain unchanged in 5x-FIRE mice, despite significant reductions in plaque intensity and insoluble amyloid levels. One of the most striking differences observed between this prior report and our current findings is that genetic loss of microglia in 5x-FIRE mice leads to a greatly shortened lifespan, with most 5x-FIRE mice dying by 6 months of age. In contrast, no changes in survival were observed in PLX5622-treated 5xfAD mice, although microglial ablation did lead to significant impairments in behavior (Spangenberg et al., 2019). Precisely why genetic microglial loss versus pharmacological ablation leads to these marked differences remains unclear. However, one possible explanation relates to the finding that long-term PLX5622 treatment leads to only a partial depletion of plaque-associated microglia within the thalamus and subiculum of 7-month-old 5xfAD mice (Spangenberg et al., 2019). Because the thalamus of 5x-FIRE mice exhibits the most robust increases in calcification, CAA, and hemorrhages, this area may be particularly vulnerable to the complete loss of microglia that occurs in FIRE mice and thalamic hemorrhages likely underlie the increased mortality observed in 5x-FIRE mice. Interestingly, Spangenberg et al. also observed a trend toward increased Prussian blue staining in PLX5622-treated 5xfAD mice, with 40% of 7-month-old mice exhibiting signs of thalamic hemorrhage.

Another possible explanation for the observed differences between PLX5622-treated 5xfAD mice and 5x-FIRE mice is that the rapid induction of apoptosis of all brain-resident microglia via CSF1R antagonists likely induces pleiotropic effects that are not present in FIRE mice. For example, it was previously shown that ASCs clear microglial corpses following CSF1R antagonist depletion, suggesting these cells alter their activation state and become hyperphagocytic (Elmore et al., 2014). Because ASCs play an integral role in BBB function, changes in astrocytic activity could provide additional protection against BBB dysfunction. Pleiotropic effects of CSF1R antagonists on peripheral immune cell populations have also been described (Lei et al., 2020), and microglial depletion has been shown to induce cytotoxic T cell infiltration into the brain (Unger et al., 2018). Given evidence that changes in T cells can influence Aβ pathology (Da Mesquita et al., 2021; Marsh et al., 2016), this presents an important yet unstudied aspect of CSF1R antagonists in the context of AD.

Another potential explanation for differences between pharmacological microglial ablation and the FIRE model relates to the potential developmental roles of microglia. Previous studies have implicated microglia and CSF1R signaling in several aspects of brain development including synaptic pruning, myelination, and neuronal survival and differentiation (Chitu and Stanley, 2017; Miron, 2017; Pridans et al., 2018; Schafer et al., 2012). Yet, FIRE mice exhibit no evidence of neurodevelopmental phenotypes (Munro et al., 2020; Rojo et al., 2019), suggesting that numerous functions attributed to microglia in nervous system development can be fulfilled by other cells (e.g., ASCs) when microglia are entirely absent. Importantly, differences between constitutive CSF1R deletion which effects many other cell types beyond microglia and the CSF1R FIRE-enhancer-specific deletion used in the current study likely underly many of the previously reported developmental abnormalities observed in traditional CSF1R knockout mice. To further address the possible existence of developmental consequences in FIRE mice, we examined the brains of 2-month-old WT-FIRE and 5x-FIRE mice, revealing no evidence of either cerebral hemorrhages or brain calcification. In addition, we performed adult microglial transplantations, providing evidence that transplantation of donor WT microglia can achieve robust engraftment within the adult mammalian brain. Most importantly, these experiments also demonstrated that delivery of donor microglia could rescue the previously observed changes in amyloid distribution, brain calcification, and cerebral hemorrhages while restoring many of the transcriptional alterations that occur in 5x-FIRE mice. Thus, we conclude it is unlikely that the observed differences between CSF1R antagonist treatments and FIRE mice are due to developmental effects.

### Limitations of the study

Important potential limitations of the current study include the use of the constitutive FIRE knockout mouse model that, despite the discussion above, may confer some as of yet unrecognized developmental effects. The use of 5xfAD mice that express high levels of Aβ42 relative to Aβ40 could also have important impacts on CAA pathology, because CAA is typically associated with a greater ratio of Aβ40/42. Lastly, given our neuropathological findings, our snRNA-seq analysis focused primarily on vascular cell types. However, this dataset also uncovers many other transcriptional changes in neurons, ASCs, and oligodendroglia when microglia are present, absent, or replaced (Figures S9–S13). Thus, we anticipate that these data will enable the formulation of exciting hypotheses regarding how microglia interact with many other CNS cell types during the progression of AD. Taken together, this study provides intriguing evidence that microglia are intimately involved in the development of multiple AD-associated pathologies. Our findings also highlight the need for additional studies that examine the impact of microglial genetics and function on vascular function, brain calcification, cerebral hemorrhages, and longevity.

## STAR★METHODS

### RESOURCE AVAILABILITY

#### Lead contact

Further information and requests for resources and reagents should be directed to and will be fulfilled by the Lead Contact, Dr. Mathew Blurton-Jones (mblurton@uci.edu).

#### Materials availability

Mouse models and induced pluripotent stem cell lines generated or used in this study will be made available on request but may require completion of a Materials Transfer Agreement.

#### Data and code availability

- Single-nuclei RNA-seq data have been deposited at GEO and are publicly available as of the date of publication under series GSE189033. Microscopy and biochemical data reported in this paper will be shared by the Lead contact upon request. Tables providing cell-specific gene expression differences between groups and WGCNA analyses can be downloaded from Mendeley https://doi.org/10.5281/zenodo.6565145). The DOI is listed in the Key resources table.
- All code used to process and analyze the SPLiT-seq dataset is publicly available on GitHub: https://github.com/swaruplabUCI/FIRE-mouse-2021-paper.
- Any additional information required to reanalyze the data reported in this paper is available from the Lead contact upon request.

### EXPERIMENTAL MODELS AND SUBJECT DETAILS

#### Mice

All animal procedures were conducted in accordance with the guidelines set forth by the National Institutes of Health (NIH) and the University of California, Irvine Institutional Animal Care and Use Committee. Equivalent numbers of both male and female mice were used and all groups were age and sex matched and group housed on a 12h/12h light/dark cycle with food and water ad libitum. Csf1r(ΔFIRE/ΔFIRE) mice were generated and previously characterized by Drs. Clare Pridans and David Hume (Rojo et al., 2019). FIRE mice were generated on a B6CBAF1/J background. Founders were then crossed with C57BL/6 mice and their offspring interbred. 5xFAD transgenic mice with C57BL/6J background overexpress both mutant human APP (695) with the Swedish (K670N, M671L), Florida (I716V), and London (V717I) Familial Alzheimer’s Disease (FAD) mutations and human PS1, harboring two FAD mutations, M146L and L286V. Expression of both transgenes is regulated by neural-specific elements of the mouse Thy1 promoter to drive overexpression in the brain. The 5XFAD-Csf1r(ΔFIRE/ΔFIRE) (5XFIRE) model was created by crossing the Csf1r(ΔFIRE/ΔFIRE) mouse with 5xFAD (+/−) mouse. Progeny of these pairings were genotyped and backcrossed with Csf1r(ΔFIRE/ΔFIRE) mice. All tissue and samples examined in this study were collected from 5–6 month old mice that showed no overt evidence of health problems at the time of sacrifice. For example, all mice were active, well groomed, and exhibited normal weight and activity. In addition, liver, lungs, and heart were examined at the time of sacrifice and all organs appeared normal. Any mice within the colony that exhibited signs of distress or illness were instead euthanized and not used for histological, biochemical, and RNA-sequencing studies. Importantly, none of the mice that were sacrificed at 5–6 months and used in the survival analyses depicted in Figure 1.

#### Human autopsy cases

De-identified fixed human prefrontal cortex tissue was obtained from the tissue repository located at the Alzheimer’s Disease Research Center (ADRC) at the University of California Irvine. Tissue collection and handling adhered to the University of California, Irvine, Institutional Review Board guidelines. Brain tissue was fixed in 4% paraformaldehyde and stored at 4°C in a solution of PBS with 0.02% sodium azide until used for histological analysis.

### METHOD DETAILS

#### Immunohistochemistry/immunofluorescence

Animals were perfused with ice-cold 1XPBS. Half brains were drop fixed in 4% PFA for 48 hours. Hemispheres were cryoprotected in 30% sucrose/1XPBS until the tissue sank in the solution. The brains were coronally sectioned at 30 µm thickness on a sliding microtome (Leica SM 2010R) cooled to −70°C. For long-term storage, free-floating sections were stored in 0.05% NaN3 and 1XPBS solution. Sections were blocked in 10% goat/donkey serum in 1 × PBS, and 0.2% Triton X-100, for 1 hour at room temperature. Amyloid Stain Reagent Amylo-Glo RTD was used before adding primary antibodies (1:100; Biosensis TR-300-AG). Overnight incubation was performed for primary antibodies followed by 1-hour secondary incubation. Sections were stained with IBA-1 (1:200, 019–19741, Wako) followed by Alexa Fluor secondary antibodies (1:400, Invitrogen). Additional samples were stained in anti-claudin-5 (1:500; 35–2500; Invitrogen), goat anti-CD13 (1:500, cat. # AF2335, R&D Systems), or AF647-RIS (1:1000; BV500101, Biovinc). Immunofluorescence-stained sections were visualized using an Olympus FV3000RS confocal microscope. Images represent confocal Z-stack taken with identical laser and detection settings. For quantification, Z-stack images were taken at ×40 magnification (10 slices taken with a Z thickness of 1 µm). The Prussian blue staining was performed using Iron stain kit (ab150674; Abcam), and Alizarin Red S (A5533, Milipore) was used for calcification labeling. Keyence BZ-X810 Widefield Microscope/Maestro Edge was used to capture these labels. Using ImageJ software boundaries of the stained area were detected and measured. n = 8 (4 Female and 4 Male) animal per genotype and condition (WT-WT, 5x-WT, WT-FIRE, 5x-FIRE, 5x-FIRE-PBS, 5x-FIRE-MG) overall n = 48 animals.

#### Flow cytometry

5–6 month-old WT-WT (n = 4), 5x-WT (n = 4), WT-FIRE (n = 4), and 5x-FIRE (n = 3) mice were sacrificed by carbon dioxide asphyxiation. Spleen, bone marrow, and deep cervical lymph nodes were harvested prior to intracardial perfusion with 0.01M phosphate buffered saline (PBS) which was followed by brain isolation without removal of the meninges. Spleens were collected in 5mL of RPMI and kept cold, manually dissociated with glass homogenizers, treated with TAC to lyse red blood cells, filtered through 70um mesh, resuspended, and stained for flow. Bone marrow was carefully extracted from the femur and tibia of the mice, treated with TAC, filtered through 70um mesh, resuspended, and stained for flow. Deep cervical lymph nodes were collected in 5mL of RPMI and kept cold, manually dissociated with glass homogenizers, filtered through 70um mesh, resuspended, and stained for flow. Brain samples were examined using the following panel of antibodies (catalog numbers provided in Star Methods): BV421-CD11b, APC/Cy7- CD45, PE-P2RY12, PE/Cy7-TMEM119. Spleen, bone marrow, and deep cervical lymph nodes were immunophenotyped with: FITC-NK1.1, PE-B220, PE/Cy7-CD45, APC/Cy7- CD8, Alexa 700- CD11b), BV421- CD3, BV510-CD11c, BV785- CD4, BV605- Gr-1, BV421- CD11b, BV711- CD45. All cells for flow cytometry were FC blocked with anti-CD16/32 (1:200) and incubated in the dark with flow antibodies for 30 minutes, followed by one rinse in FACS buffer (5 min, 4°C, 1400 rpm). Cells were then examined on a Fortessa flow cytometer (BD Biosciences) and analyzed with FlowJo V10.8 software (FlowJo; Ashland, OR) using Fluorescence minus one (FMO) controls to establish gating strategies.

#### Intracranial transplantation

Bilateral intracranial injections of primary mouse microglia into the cortex and hippocampus were performed on 2-month-old 5x-FIRE mice (n = 4 female and n = 4 male). All mouse surgeries and use were performed in strict accordance with approved NIH and AALAC-certified institutional guidelines. Mice were briefly anesthetized under continuous isoflurane and positioned into a stereotaxic frame (Kopf). Mice were disinfected with alcohol and iodine before and after shaving the head area, and the anesthetic (Lidocaine 2%) was applied to the head area before exposing the skull. Primary mouse microglia (ScienCell, Catalog #M1900–57) were thawed into microglial media (ScienCell, Catalog #1901) and an aliquot of cells was examined by flow cytometry for the homeostatic microglia markers P2RY12 and TMEM119. Remaining microglia were placed on ice and used for transplantation within an hour of thawing. Blood derived murine monocytes (Cell Biologics, Catalog #C57–6271F) were similarly thawed and used as a negative control for flow cytometry assessment (Figure 3B). Within an hour of thawing, microglia were collected via centrifugation and resuspended in sterile 1XPBS at 40,000 cells/µL. Using a 30-gauge needle affixed to a 10µL Hamilton syringe, 2µL of microglial suspension was administered bilaterally. The cells were injected at a rate of 1 µL/30 s with a 4-minute delay between injections. Each mouse received a total of 160,000 cells, which were bilaterally transplanted in the hippocampus and cortex, relative to bregma: anteroposterior, −2.06 mm; mediolateral, ± 1.75 mm; dorsal-ventral, −1.75 mm (hippocampus) and −0.95 mm (cortex). The identical approach was used to deliver an equivalent volume of PBS vehicle for control mice (n = 4 female and n = 4 male). Following the surgery, mice received 2 mg/mL acetaminophen diluted in water for five days. All mice were then sacrificed at 5 months of age. Shorter term (24 day) pilot studies to examine repopulation kinetics were also performed using an equivalent unilateral stereotactic approach and transplantation of 80,000 microglia (Figures 3H–3N).

#### MSD/ELISA

Half brain hemispheres, that were stored at − 80°C, were crushed on dry ice using mortar and pestle, then homogenized in T-PER solution (Thermo Scientific, Waltham, MA) and phosphatase and protease inhibitor mixtures (Thermo Scientific, MA and Roche, CA) and processed as previously described (Marsh et al., 2016). Quantitative biochemical analysis of human Aβ was conducted using commercially available electro-chemiluminescent multiplex assay system [Meso Scale Discovery (MSD)]. Human Aβ duplex (6E10 capture antibody) was used for simultaneous measurement of Aβ40 and Aβ42 in both soluble and insoluble protein fractions (Marsh et al., 2016).

#### iPSC-microglia differentiation

iPSC-microglia were generated as described (McQuade et al., 2018). Briefly, iPSCs were directed down a hematopoietic lineage using the STEMdiff Hematopoesis kit (StemCell Technologies). After 10–12 days in culture, CD43^+^ hematopoteic progenitor cells are transferred into a microglia differentiation medium containing DMEM/F12, 2× insulin-transferrin-selenite, 2× B27, 0.5× N2, 1× glutamax, 1× non-essential amino acids, 400 µM monothioglycerol, and 5 µg/mL human insulin. Media was added to cultures every other day and supplemented with 100 ng/mL IL-34, 50 ng/mL TGF-β1, and 25 ng/mL M-CSF (Peprotech) for 28 days. In the final 3 days of differentiation 100 ng/mL CD200 (Novoprotein) and 100 ng/mL CX3CL1 (Peprotech) were added to culture.

#### CRISPR-mediated knockout of *TREM2*

Generation of isogenic T*REM2* knockout iPSC lines was previously described (McQuade et al., 2020). Briefly, iPSCs were nucleofected with RNP complex targeting the second exon of *TREM2* and allowed to recover overnight. Transfected cells were dissociated with pre-warmed Accutase then mechanically plated to 96-well plates for clonal expansion. Genomic DNA from each colony was amplified and sequenced at the cut site. The amplification from promising clones was transformed via TOPO cloning for allelic sequencing. Knockout of *TREM2* was validated by western blotting (AF1828, R&D) and HTRF (Cisbio) (McQuade et al., 2020).

#### Phagocytosis of hydroxyapatite

Hydroxyapatite (HAp <200 nm) (Sigma; 677418) were diluted in DMSO (10 mM) and stained 1:400 with AF647-RIS (Biovinc; BV500101) for 1 hr at room temperature. After staining, HAp was washed with excess DBPS three times (centrifugation at 16,000 xG for 1 min) prior to use. iPSC-microglia expressing cytoplasmic GFP (Coriell; WTC11 background) were differentiated as described above and plated at 70% confluence on Matrigel-coated glass 96-well plates for 24 hrs prior to exposure to 100 uM pre-stained HAp. No toxicity was observed over 24 hrs via Caspase 3/7 reagent (IncuCyte; 4440). After 24 hours, iPSC-microglia were fixed with 4% paraformaldehyde for 7 min and washed 3x with excess DPBS. Cells were counterstained with Hoechst.

#### Western blot

Soluble brain fractions were prepared by homogenizing mouse brain powder in Protein Extraction Buffer containing Tissue Protein Extraction Reagent, protease and phosphatase inhibitors, and EDTA (Thermo Fisher Scientific). Samples were centrifuged at 16,000g for 5 minutes then the supernatant was collected, and protein concentration was measured using a Pierce BCA Assay Kit (Thermo Fisher Scientific). Sample volumes containing 20ug of protein were mixed with reduction buffer containing 2X Laemmli Sample Buffer (BioRad) and 5% beta-mercaptoethanol (Millipore Sigma) and heated at 95°C for 10 minutes. Samples were run on a 4–15% polyacrylamide gel in Tris/Glycine/SDS buffer (BioRad) and transferred to a nitrocellulose membrane (BioRad). The membrane was blocked in 5% BSA in TBST and probed with primary antibodies at the following concentrations overnight at 4°C: Either goat anti-Human APP (1:1000, AF1168, R&D Biosystems) rabbit anti-IBA-1 (1:500, GTX100042, GeneTex) or mouse anti-beta-Actin (1:2,000, A2228, Millipore Sigma). The following day, the membrane was washed and probed with either HRP-conjugated anti-goat (1:5,000, AP180P, Millipore Sigma), anti-Rabbit (1:5000, AP106P, Millipore Sigma) or anti-mouse secondary antibodies (1:5000, AP180P, Millipore Sigma) for 1 hour at room temperature. After washing, the membrane was imaged with SuperSignal West Dura Extended Duration Substrate (Thermo Fisher Scientific) using the BioRad Molecular Imager ChemiDoc XRS with Image Lab Software. The membrane was stripped and re-blocked between each protein staining and all steps were carried out with gentle agitation. Analysis was carried out using ImageJ software.

#### Single-nucleus RNA-sequencing (SnSeq)

Single nucleus suspensions were generated from5–6 month old mice (n = 48, 6 groups, 4 female 4 male) as previously described (Chen et al., 2013; Morabito et al., 2020, 2021) except after the 40µm filtration, nuclei were immediately processed using the Nuclei Fixation kit (Parse Biosciences, formerly Split Biosciences) and then cryopreserved with DMSO until library preparation. Nuclei isolation was performed in randomized groups of eight samples, while library preparation was performed in a single batch of all 48 samples. Single-nucleus RNA-seq libraries were generated with the Single Cell Whole Transcriptome library preparation kit (Parse Biosciences), which utilizes split-pool ligation-based transcriptome-sequencing (SnSeq) as described in in (cite Rosenberg et al., 2018 Science). Briefly, each sample, normalized by nuclei number, was added to one well of a 96-well plate to perform reverse transcription, adding a well-specific barcode (first barcode). All the wells were then pooled together and randomly distributed across a new 96-well plate with new well-specific barcodes (second barcode) for ligation. After ligating the second barcode, the wells are again pooled and randomly distributed across another 96-well plate for a third round of barcoding. Throughout this process, the nuclei are still intact but permeabilized to allow the reactions to append three different barcodes to each transcript. The nuclei were then pooled into 8 sublibraries of 12,500 nuclei and lysed to allow the construction of the final cDNA libraries, in which a fourth, sublibrary-specific barcode is added. This thus results in single-nucleus transcriptomes that can be identified based up on the combination of the four different barcodes. cDNA library quality was assessed using the Agilent 4200 Tapestation, and concentrations were determined using Qubit. Libraries were sequenced using the Illumina NovaSeq 6000 S4 at UCI’s Genomics High-Throughput Facility for a sequencing depth of 50,000 read pairs/cell.

#### SnSeq read processing, quality control and filtering

Gene expression was quantified using the split-pipe software (v0.7.7p, Parse Biosciences). First, sequences were downloaded and gene annotation comprising the mouse reference transcriptome (GRCm38.93, Ensembl), and formatted using the function split-pipe mkref. Next, SnSeq reads were aligned and gene expression quantified (STAR (Dobin et al., 2013), v2.7.8a) using split-pipe. This process was repeated for eight SnSeq sub-libraries, resulting in eight gene expression matrices, which was then merged using the Matrix package (v1.2.18) in R (v3.6.3). Finally, a Seurat (Butler et al., 2018; Stuart et al., 2019) (v3.2.2) object was constructed for the downstream analysis of this merged gene expression matrix of all 48 experimental samples. Nuclei with greater than 5% of reads mapped to mitochondrial genes and greater than 20,000 total UMIs were removed. The lowest total UMI value in a single nucleus was 1,389, therefore nuclei were not filtered out based on low UMI values. DoubletFinder (McGinnis et al., 2019) (v2.0.3) was used to predict events where one barcode was assigned to multiple nuclei, and 7.5% of nuclei were removed that were confidently predicted as doublets. In total, 31,746 nuclei were filtered, retaining 51,327 for downstream analysis.

#### Dimensionality reduction and clustering

The gene expression matrix was normalized and scaled using the Seurat functions NormalizeData and ScaleData respectively. The Seurat function FindVariableFeatures was used to identify the most variable genes, of which the top 3,500 were used for dimensionality reduction. Principal Component Analysis (PCA) was used to reduce the dimensionality of the scaled gene expression matrix, and the first 30 PCs were harmonized by experimental sample using the R package Harmony (Korsunsky et al., 2019) (v1.0). The harmonized PCA representation was used to construct a neighborhood graph using the sc.pp.neighbors function from the Python (v3.7.9) package SCANPY (Wolf et al., 2018) (v1.6.0) with n = 15 neighbors and cosine distance. Nuclei were grouped into clusters using the leiden (Traag et al., 2019) clustering algorithm with the SCANPY function sc.tl.leiden and cluster resolution = 1.5. A cluster-level graph of the data was generated by performing Partition-based graph-abstraction (PAGA) (Wolf et al., 2019)) with the scanpy function sc.tl.paga, where each node represents a cluster and weighted edges quantify connectivity between clusters. A two-dimensional representation of gene-expression space was constructed using the Uniform Manifold Approximation and Projection (McInnes et al., 2018) (UMAP) algorithm with the SCANPY function sc.tl.umap, using the PAGA graph for initialization.

#### Cluster annotation and comparison with SnSeq data from the mouse brain

Cell-type annotations were assigned to each cluster based on two levels of evidence. First, the Seurat function FindAllMarkers was used to identify cluster marker genes based on one-versus-all Wilcoxon rank sum differential expression tests for each cluster. Next, the cell-type identity of all nuclei was predicted based on a published SnSeq dataset of the mouse brain (Rosenberg et al., 2018). Anchor points between the labeled reference dataset and our own dataset were identified using the Seurat function FindTransferAnchors, and cell-type prediction scores were computed for all nuclei based on the 73 annotations present in the reference dataset. The most likely predicted cell-type annotations were cross-referenced with the marker genes for that cluster to give a cell-type annotation for each cluster. Furthermore, clusters were manually grouped into major cell classes, for example merging excitatory neurons from different cortical layers and brain regions into one excitatory cell class. Cell-types without any sub-clusters, such as ependymal cells, were not grouped into larger cell lineages.

### QUANTIFICATION AND STATISTICAL ANALYSIS

Data are presented as SEM and n represents the number of technical replicates of cell culture experiments or animal numbers, unless specified. Most statistical comparisons were conducted by ANOVA for Bonferroni’s post hoc test in GraphPad Prism 9 and significant difference was defined as p < 0.05 unless specified. Image processing and quantification was performed using IMARIS software and for intensity and bright filed images, ImageJ software was used as detailed below.

#### Differential gene expression analysis comparing experimental conditions

Gene expression signatures of different experimental conditions within each cell class were compared using the Seurat function FindMarkers using a Wilcoxon rank-sum test. The following comparisons were performed: 5XFAD vs. 5xFIRE; 5XFIRE vs 5XFIRE + transplanted microglia; 5XFIRE + transplanted microglia vs 5XFIRE + PBS. EnrichR (Kuleshov et al., 2016) (v 3.0) was used to test for gene sets enriched in up- and down-regulated genes (top 75 by fold-change, false-discovery rate less than or equal to 0.1) in the following databases: GO Biological Process_2018, GO Cellular Component 2018, GO Molecular Function 2018, Wikipathways Mouse 2019, KEGG Mouse 2019.

#### Analysis of disease-associated microglia (DAM) gene expression signatures

Using the Seurat function AddModuleScore, gene module scores were computed for disease-associated microglia signatures (Keren-Shaul et al., 2017) and a homeostatic microglia signature using the following gene lists: stage 1 *Trem2* independent DAM markers (*Tyrobp*, *Ctsb*, *Apoe*, *B2m*, *Fth1*); stage 2 *Trem2* dependent DAM markers (*Trem2*, *Axl*, *Cst7*, *Ctsl*, *Lpl*, *Cd9*, *Csf1*, *Itgax*, *Clec7a*, *Timp2*); homeostatic microglia markers (*Hexb*, *Cst3*, *Cx3cr1*, *Ctsd*, *Csf1r*, *Ctss*, *Sparc*, *Tmsb4x*, *P2ry12*, *C1qa*, *C1qa*). The distributions of gene module scores were compared within the immune cell class between experimental conditions using a Wilcoxon rank-sum test.

#### Single-cell weighted gene co-expression network analysis

Single cell Weighted Gene Co-expression Network Analysis (scWGCNA) was performed using the scWGCNA R package (v 0.0.0.9000). Log-normalized gene expression of nuclei within each cell class and experimental condition were aggregated using the construct_metacells function from the scWGCNA package. To check if the aggregated expression profiles retain a similar level of cellular heterogeneity as in the full dataset, dimensionality reduction analysis of the metacell dataset was performed with the following Seurat workflow: FindVariableGenes with 1,000 variable genes, ScaleData RunPCA, RunHarmony to harmonize by experimental group, and RunUMAP with the first 15 harmonized PCs. Co-expression networks were analyzed using the R package WGCNA (v1.69) within the following selected cell groups with the metacell expression matrix as input: astrocytes, excitatory neurons, inhibitory neurons, oligodendrocyte lineage, and vasculature cells (pericytes and endothelial cells). First, the optimal soft power threshold parameter was exmained using the WGCNA function pickSoftThreshold using a signed network and bicorrelation as the correlation function, testing every other power threshold value between 1 and 30. The following soft power threshold values was selected based on the scale-free topology model fit for each cell group: ASC = 8, EX = 8, INH = 8, VASC = 9, ODC = 10. Next, an unsigned topological overlap matrix and identified co-expression modules was assembled using the WGCNA function blockwiseConsensusModules with the selected soft power threshold parameter, and the following parameters: deepSplit = 4, minModuleSize = 50, mergeCutHeight = 0.2, corType = ‘‘pearson’’. Module eigengenes (first principal component of the gene expression matrix for genes within module) were computed for all modules using the WGCNA function moduleEigengenes. Intramodular connectivity (kME) of genes within each module were computed using the WGCNA function signedKME. Gene sets enriched in each co-expression module were tested using the EnrichR package in the following databases: GO Biological Process_2018, GO Cellular Component 2018, GO Molecular Function 2018. To check that gene expression signatures of modules could be identified in the full SnSeq dataset, the Seurat function AddModuleScore was used to compute a gene module score the top 25 hub genes from each module by kME, and the resulting score was visualized using a the R package ComplexHeatmap (Gu et al., 2016) (v2.7.6.1010).

#### Cellular signaling analysis

Intercellular signaling network analysis was performed using the R package CellChat (Jin et al., 2020) (v1.0.0). Separate CellChat objects were created for all six experimental conditions, grouping by cell classes and using the CellChatDB mouse database for ligand-receptor interactions. The protocol from the CellChat github was followed to process the data and compute communication probabilities for each pathway and to construct cell signaling networks. The CellChat function netVisual aggregate was used to view the cell communication probabilities between each cell class for *Pdgf* and *Tgfb* signaling pathways.

#### IMARIS quantification of microglial density

For quantification of IBA-1 immunoreactive microglial density from confocal z stack images we utilized Bitplane IMARIS 9.8.0. As IBA-1 also labels border-associated macrophages (BAMs) including meningeal and perivascular macrophages, we therefore utilized IMARIS’s ‘‘modeling’’ and ‘‘filaments’’ features to identify IBA-1+ cells that exhibit processes emanating from the cell body. The diameter of the thinnest filament attached to the cell body was chosen as 0.6 µm, and the diameter of the filament at its starting point was set to be less than 10 μm in diameter (‘‘the soma’’). IMARIS software was then used to automatically detect all microglia in each image. The outcome of the autodetection was manually reviewed for accuracy and any inaccurate detections were notified to the software to further optimize the selection algorithm prior to data collection. After confirming that this method accurately detects all microglia within a given field but does not detect BAMs, all images were examined blinded to genotype and treatment using IMARIS batch processing. All parameters of the identified microglia (count, area, average number of branches, branch complexity, average branch length, average branch area, microglia sphericity, etc.) were saved in a csv file for further analysis. n = 8 (4 Female and 4 Male) animal per genotype and condition (PBS vs microglia transplanted) were examined.

#### IMARIS quantification of amyloid plaques and CAA

To distinguish between Aβ plaques and CAA, Amylo-Glo staining was classified using three parameters independently: length, area, and sphericity (Figure S4A). Amylo-Glo was detected using the IMARIS ‘‘Surface’’ option and thresholds used for the automated analysis were determined by visual inspection. In the first classification, surfaces with length ≥40 µm were classified as CAA and <40 µm was assigned as a parenchymal plaque. In the second classification, surfaces with sphericity ≥0.7 µm were assigned as parenchymal plaques and these data were also used to compare plaque sphericity between groups. In the third classification, surfaces with area ≥1800 µm^2^ were considered CAA and <1800 µm^2^ were assigned as parenchymal plaques. The parenchymal plaque surface was further categorized into ‘‘small’’ with a length <20 µm and ‘‘large’’ with length between 20µm and 40 µm.

## Supplementary Material

1

## Figures and Tables

**Table:** KEY RESOURCES TABLE

REAGENT or RESOURCE	SOURCE	IDENTIFIER
Antibodies		
Alexa Fluor 488 goat anti-chicken	Life Technologies	Cat #A11039; RRID: AB_2534096
Alexa Fluor 555 goat anti-mouse	Life Technologies	Cat #A21424; RRID: AB_141780
Alexa Fluor 555 goat anti-rabbit	Life Technologies	Cat #A21429; RRID: AB_2535850
Alexa Fluor 633 goat anti-mouse	Life Technologies	Cat #A21052; RRID: AB_2535719
Alexa Fluor 633 goat anti-rabbit	Life Technologies	Cat #A21071; RRID: AB_2535732
Goat anti-CD13	R&D Systems	Cat #AF2335; RRID: AB_2227288
Anti-Iba, Rabbit	Wako	Cat #019–19741; RRID: AB_839504.
Anti-claudin-5	Invitrogen	Cat #35–2500; RRID: AB_2533200
Perivascular macrophages (PVMs)-CD206	Thermo Fisher Scientific	Cat #PA5–46994; RRID: AB_2607366
Anti-Human APP	R&D Biosystems	Cat #AF1168; RRID: AB_354642
Mouse anti-beta-Actin	Millipore Sigma	Cat #A2228; RRID: AB_476697
Rabbit anti-IBA-1	GeneTex	Cat #GTX100042, RRID: AB_1240434
LYVE1 Monoclonal Antibody	ThermoFisher	Cat #14–0443-82; RRID: Cat #14–0443-82
HRP-conjugated anti-goat	Millipore Sigma	Cat #AP180P; RRID: AB_92573
Goat anti-Mouse IgG (H+L) Secondary Antibody, HRP	ThermoFisher	Cat # 62–6520; RRID: AB_2533947
Anti-Rabbit secondary antibodies	Millipore Sigma	Cat #AP106P; RRID: AB_92411
PE-P2RY12	BioLegend	Cat #84003; RRID: AB_11028449
PE/Cy7-TMEM119	ThermoFisher	Cat #25–6119-82; RRID: AB_2848312
BV421-CD11b	BioLegend	Cat #101235; RRID: AB_10897942
FITC-NK1.1	BioLegend	Cat #156508; RRID: AB_2876526
PE-B220	BioLegend	Cat #103207; RRID: AB_312992
PE/Cy7-CD45	BioLegend	Cat #103114; RRID: AB_312978
APC/Cy7-CD8	BioLegend	Cat #100714; RRID: AB_312752
Alexa 700-CD11b	BioLegend	Cat #101222; RRID: AB_493705
BV421-CD3	BioLegend	Cat #100228; RRID: AB_10900227
BV510-CD11c	BioLegend	Cat #117353; RRID: AB_2562010
BV785-CD4	BioLegend	Cat #100552; RRID: AB_11218992
BV605-Gr-1	BioLegend	Cat # 108439; RRID: AB_2562333
BV421-CD11b	BioLegend	Cat #101236; RRID: AB_10897942
BV711-CD45	BioLegend	Cat #304050; RRID: AB_2563465
anti-CD16/32	BD Biosciences	Cat #56–0161-82; RRID: AB_493994
Biological samples		
UCI ADRC Brain Bank	https://mind.uci.edu/adrc/neuropathology-core/	Case Numbers: 39–10, 4–14, 1–8, 29–15, 3–12, 38–14, 8–18, 22–17, 51–15, 9–18, 35–17
Primary mouse microglia	ScienCell	Cat #M1900–57
C57BL/6 Mouse Bone Marrow Monocytes	CellBiologics	Cat # C57–6271F
Chemicals, peptides, and recombinant proteins		
Triton X-100	Fisher Scientific	Cat #BP151–500
Paraformaldehyde	Sigma-Aldrich	Cat #P6148–500G
Sodium Azide	Sigma-Aldrich	Cat #S8032–100G
Goat serum	Thermo Fisher Scientific	Cat #10000C; RRID: AB_2532979
Amylo-Glo RTD Amyloid Plaque Stain Reagent	Biosensis	Cat #TR-300-AG
DAPI Fluoromount-G	SouthernBiotech	Cat #0100–20
Fluoromount-G	SouthernBiotech	Cat #0100–01
Ethyl alcohol	Pure Sigma Aldrich	Cat #E7023
2-propanol	Sigma Aldrich	Cat #59304
Chloroform:Isoamyl alcohol	Sigma Aldrich	Cat #C0549
Hydroxyapatite (HAp <200 nm)	Sigma Aldrich	Cat #677418
SuperSignal West Dura Extended Duration Substrate	Thermo Fisher Scientific	Cat #34075
Pierce BCA Assay Kit	Thermo Fisher Scientific	Cat #23225
Laemmli Sample Buffer	BioRad	Cat #1610747
Beta-mercaptoethanol	Millipore Sigma	Cat #M6250
AF647-RIS	Biovinc	Cat #BV500101
Iron stain kit	Abcam	Cat #ab150674
Alizarin Red S	Milipore	Cat #A5533
4–15% polyacrylamide gel in Tris/Glycine/SDS buffer	BioRad	Cat #5671084
0.2µm Nitrocellulose membrane	BioRad	Cat# #1704159
T-PER solution	Thermo Scientific	Cat #78510
Hoeschst 33342	ThermoFisher Scientific	Cat #R37165
Monothioglycerol	Sigma Aldrich	Cat #M6145
Phosphatase and protease inhibitor mixtures	Thermo Scientific	Cat #78440
Caspase 3/7 reagent	Essen Biosciences	Cat #4440
Insulin-Transferrin-Selenium (ITS-G)	Thermo Fisher Scientific	Cat #41400045
Human Insulin	Sigma Aldrich	Cat #I2643
Recombinant Human IL-34	Peprotech	Cat #200–34
Recombinant Human M-CSF	Peprotech	Cat #300–25
Recombinant Human TGF-b1 (HEK293 derived)	Peprotech	Cat #100–21
Recombinant Human Fractalkine (CX3CL1)	Peprotech	Cat #300–18
Recombinant Human OX-2/MOX1/CD200 (C-6His)	Novoprotein	Cat #C311
ReLeSR	StemCell Technologies	Cat #05872
BD Matrigel Matrix Growth Factor Reduced, PhenolRed-Free	BD Biosciences	Cat #356231
Thiazovivin	StemCell Technologies	Cat #72252
BamBanker	Wako	Cat #NC9582225
1X DPBS, no Ca^2+^, no MG2+	Thermo Fisher Scientific	Cat #14190250
10X HBSS, no Ca^2+^, no MG2+, no phenol red	Thermo Fisher Scientific	Cat #14185052
1X HBSS, no Ca^2+^, no MG2+, no phenol red	Thermo Fisher Scientific	Cat #14025126
MACS BSA Stock Solution	Miltenyi Biotech	Cat #130–091-376
Critical commercial assays		
Nuclei Fixation Kit	Parse Biosciences	Cat #SB1003
Single Cell Whole Transcriptome - 100k cells/nuclei, up to 48 samples	Parse Biosciences	Cat #SB1003
Barcoding - Whole Transcriptome	ParseBiosciences	Cat #SB1010
Library Prep - Whole Transcriptome	Parse Biosciences	Cat #SB1011
Nuclei EZ Lysis Buffer	Millipore Sigma	Cat # NUC101–1KT
RNase Inhibitor	Fisher Scientific	Cat #M0314L
Sucrose cushion buffer	Sigma-Aldrich	Cat #NUC-201
Direct-zol RNA Miniprep Plus Kits	ZYMO Research	Cat #R2071
STEMdiff Hematopoiesis kit	StemCell Technologies	Cat #05310
Povidone Iodine	Phoenix	Cat #36LF58
Lidocaine Hydrochloride Jelly	Akorn	Cat #NDC17478–711-30
Lidocaine Hydrochloride Injectable	Phoenix	Cat #PHO5731909305
Vetbond	3M	Cat # 014006
Sterile Saline	CareFusion	Cat # AL4109
Isoflurane	Patterson Veterinary	Cat #14043070406
Sterile Artificial Tears Ointment	Rugby	https://www.chewy.com/akorn-artificial-tears-lubricant/dp/315333
Deposited data and code		
SPLiT sequencing datasets	This study	GEO series #GSE189033
Tables providing cell-type specific gene expression differences and WGCNA analyses	This study	Mendeley https://doi.org/10.5281/zenodo.6565145
Code used to process and analyze SPLiT-seq dataset	This study	https://doi.org/10.5281/zenodo.6565145
Experimental models: Cell lines		
mono-allelic mEGFP-tagged WTC11 Ipsc line	Coriell	Cat# AICS-0036–006
TREM2 CRISPR knockout on mEGFP-tagged WTC11 iPSC	Blurton-Jones lab	see McQuade et al., 2020
Experimental models: Organisms/strains		
Mouse: 5X-FAD; B6.Cg-Tg(APPSwFlLon,PSEN1*M146L*L286V) 6799Vas/Mmjax	The Jackson Laboratory	Jax stock no. 34848-JAX
Csf1rDFIRE/DFIRE *C57BL/6	David A. Hume & Clare Pridans	See Rojo et al., 2019
Mouse: 5x-FIRE cross:	This paper	5x-FIRE mice
Software and algorithms		
GraphPad Prism 9	GraphPad	RRID:SCR_002798
ImageJ	NIH	RRID:SCR_003070
FlowJo software V10.8	FlowJo; Ashland, OR	RRID:SCR_008520
Imaris (9.2.0)	Imaris	RRID:SCR_007370
BioRender	BioRender	RRID:SCR_018361
Split-pipe software (v0.7.7p, Parse Biosciences)	Parse Biosciences	Split-pipe software (v0.7.7p)
R (v3.6.3)	https://cran.r-project.org/web/packages/Matrix/index.html	RRID:SCR_001905
Seurat (v3.2.2)	https://www.r-project.org/	RRID:SCR_016341
DoubletFinder (v2.0.3)	Stuart et al., 2019	RRID:SCR_018771
Harmony (v1.0)	McGinnis et al., 2019	RRID:SCR_022206
Python (v3.7.9)	https://www.python.org	RRID:SCR_008394
SCANPY (v1.6.0)	Wolf et al., 2018	RRID:SCR_018139
scWGCNA R package (v 0.0.0.9000)	Morabito et al., 2021	https://github.com/smorabit/scWGCNA
WGCNA (v1.69)	Langfelder and Horvath, 2008	RRID:SCR_003302
ComplexHeatmap (v2.7.6.1010)	Gu et al., 2016	RRID:SCR_017270
Enrichr	Chen et al., 2013, Kuleshov et al., 2016	RRID:SCR_001575
CellChat (v1.0.0)	Jin et al., 2021	RRID:SCR_021946
Other		
Olympus FV3000RS confocal microscope	Olympus	https://www.olympus-global.com/news/2016/nr160405fv3000e.html
Keyence BZ-X810	Keyance	https://www.keyence.com/products/microscope/fluorescence-microscope/bz-x700/models/bz-x810/
Fortessa flow cytometer	BD Biosciences	https://www.bdbiosciences.com/en-eu/products/instruments/flow-cytometers/research-cell-analyzers/bd-lsrfortessa
6-well plates	Corning	Cat #3516
Microscope slides	VWR	Cat #16004–392
Coverslips	Fisher Scientific	Cat #12–548-5M
Masterflex L/S Perfusion Pump	Masterflex L/S Perfusion Pump	https://www.masterflex.com/collections/masterflex-ls-complete-pump-systems
10uL Gastight syringe, Model 1701 RN	Hamilton	Cat #7653–01
30-gauge, Small hub RN needle, 12mm, Pt:4, 45 tip	Hamilton	Cat #7803–07
Germinator 500 Dry Sterilizer	Cell Point Scientific	Cat #GER5287120V
Sliding microtome	Leica SM 2010R	Leica SM 2010R
Agilent 4200 Tapestation	Agilent	Part Number:G2991BA
Illumina NovaSeq 6000 S4	Illumina	https://www.illumina.com/systems/sequencing-platforms/novaseq.html
BioRad Molecular Imager ChemiDoc XRS	BioRad	ChemiDoc™ XRS+ System with Image Lab™ Software #1708265

## Highlights

- Absence of microglia in Alzheimer’s disease mice promotes cerebral amyloid angiopathy
- Increased vascular amyloid is accompanied by hemorrhages, calcification, and lethality
- Adult transplantation of microglia prevents these diverse pathological changes
- snRNA-seq reveals the impact of microglial absence and replacement on other cell types
