# Supplementary material for: Absence of microglia promotes diverse pathologies and early lethality in Alzheimer’s disease mice
Source: Cell Rep. Author manuscript; Available in PMC 2022 Jul 15. (PMC9285116; doi:10.1016/j.celrep.2022.110961)
Supplement: 1 [file NIHMS1816595-supplement-1.pdf]

Cell Reports, Volume *39*

## Supplemental information

# Absence of microglia promotes diverse pathologies and early lethality in Alzheimer's disease mice

**Sepideh Kiani Shabestari, Samuel Morabito, Emma Pascal Danhash, Amanda McQuade, Jessica Ramirez Sanchez, Emily Miyoshi, Jean Paul Chadarevian, Christel Claes, Morgan Alexandra Coburn, Jonathan Hasselmann, Jorge Hidalgo, Kayla Nhi Tran, Alessandra C. Martini, Winston Chang Rothermich, Jesse Pascual, Elizabeth Head, David A. Hume, Clare Pridans, Hayk Davtyan, Vivek Swarup, and Mathew Blurton-Jones**

Figure S1

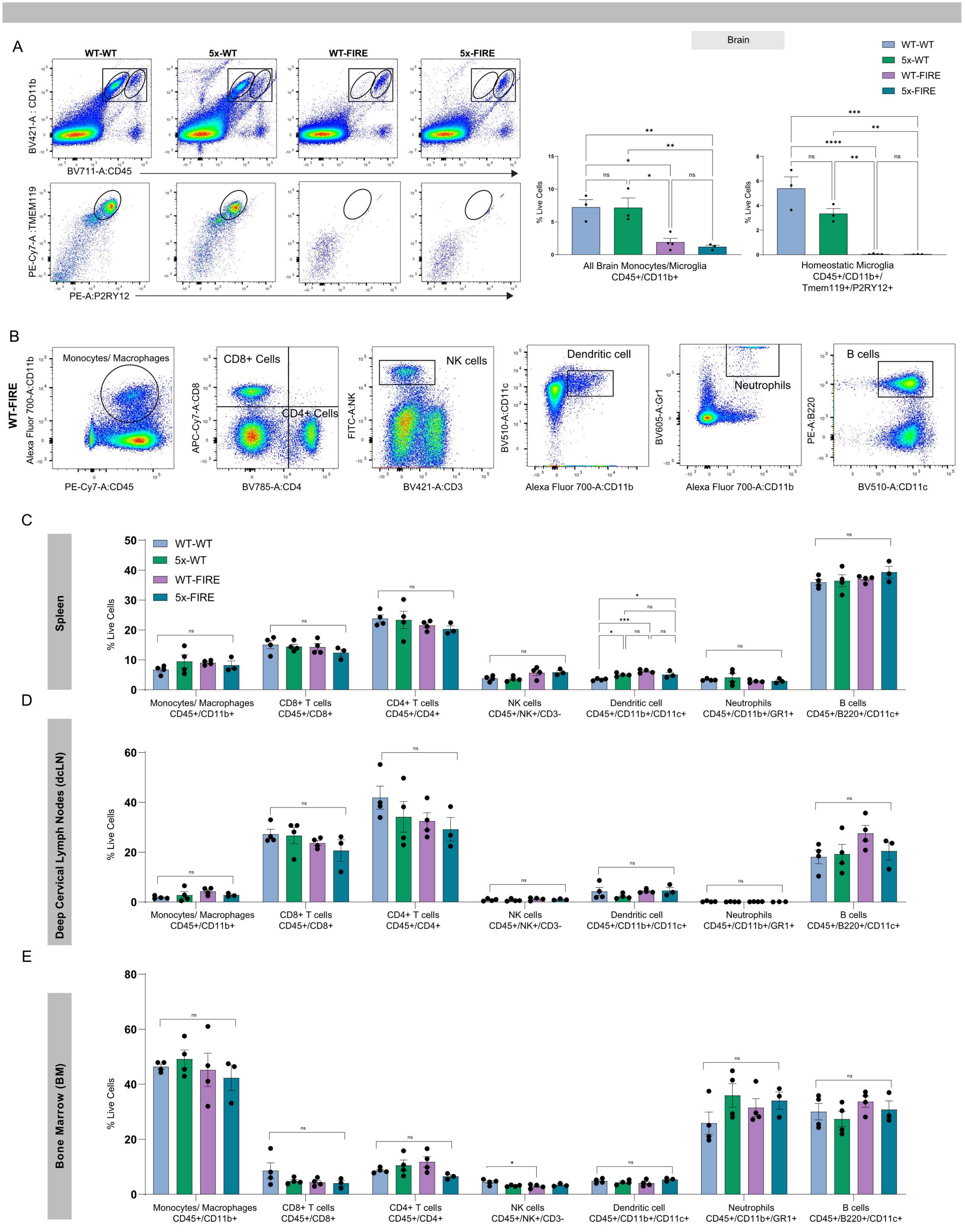

A
WT-WT
5x-WT
WT-FIRE
5x-FIRE
BV421-A : CD11b
BV711-A:CD45
PE-Cy7-A :TMEM119
PE-A:P2RY12
Brain
% Live Cells
All Brain Monocytes/Microglia
CD45+/CD11b+
Homeostatic Microglia
CD45+/CD11b+/
Tmem119+/P2RY12+
B
WT-FIRE
Monocytes/ Macrophages
Alexa Fluor 700-A:CD11b
PE-Cy7-A:CD45
CD8+ Cells
CD4+ Cells
APC-Cy7-A:CD8
BV785-A:CD4
NK cells
FITC-A:NK
BV421-A:CD3
Dendritic cell
BV510-A:CD11c
Alexa Fluor 700-A:CD11b
Neutrophils
BV605-A:Gr1
Alexa Fluor 700-A:CD11b
B cells
PE-A:B220
BV510-A:CD11c
C
Spleen
D
Deep Cervical Lymph Nodes (dcLN)
E
Bone Marrow (BM)
% Live Cells
Monocytes/ Macrophages
CD45+/CD11b+
CD8+ T cells
CD45+/CD8+
CD4+ T cells
CD45+/CD4+
NK cells
CD45+/NK+/CD3-
Dendritic cell
CD45+/CD11b+/CD11c+
Neutrophils
CD45+/CD11b+/GR1+
B cells
CD45+/B220+/CD11c+

**Supplemental Figure 1: Flow cytometry confirms the loss of microglia in FIRE mice and very few other changes to immune cell populations.** Related to Figure 1. **(A)** To further confirm that FIRE mice exhibit a loss of microglia flow cytometry was performed. Initial gating for CD45 and CD11b double-labeled cells reveals two distinct populations (ovals within boxed region). CD11b+/CD45int is commonly used to identify microglia and is greatly reduced in both FIRE groups. Subsequent gating of cells for the homoeostatic microglia markers P2RY12 and TMEM119 further confirmed that microglia are absent in FIRE mice, consistent with our histological and snSeq findings. **(B)** As CSF1R is also expressed in some peripheral immune lineages we next examined the proportions of major immune cell types using a panel of immunophenotyping markers. For each cell type examined we detected no differences in the percentage of immune cells across all four genotypes within the Spleen **(C)**, deep cervical lymph nodes **(D)**, and bone marrow **(E)**, with one notable exception. Dendritic cells (CD45+/CD11b+/CD11c+) exhibited a small but significant increase in 5x-WT, WT-FIRE, and 5x-FIRE groups in comparison to WT-WT mice (C). All graphs presented as mean ± s.e.m.

Figure S2

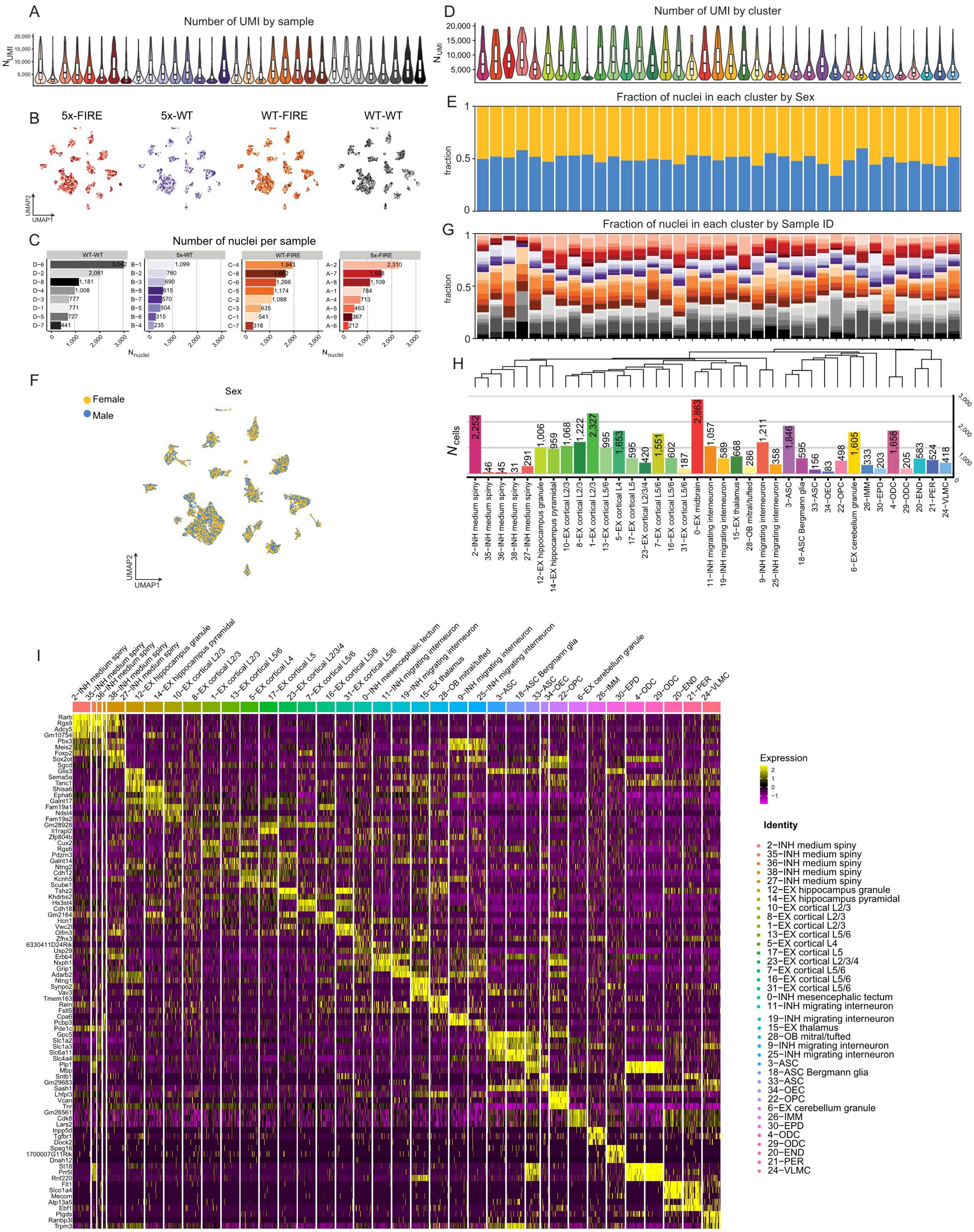

A
Number of UMI by sample
D
Number of UMI by cluster
B
5x-FIRE
5x-WT
WT-FIRE
WT-WT
E
Fraction of nuclei in each cluster by Sex
C
Number of nuclei per sample
G
Fraction of nuclei in each cluster by Sample ID
F
Sex
Female
Male
H
I
Expression
Identity

**Supplemental Figure 2: snRNA-seq data quality.** Related to Figure 1. **(A)** Violin plots showing the distribution of the number of UMIs captured in each biological for the 5x-FIRE, 5xFAD, WT-FIRE, and WT-WT conditions. **(B)** UMAP plots colored split by cells from each of the four genotypes and colored by sample of origin. **(C)** Bar plot reporting the number of nuclei recovered in each sample in each genotype. **(D)** Violin plots showing the distribution of the number of UMIs for each cluster. **(E)** Stacked bar plot showing the normalized proportion of nuclei from male (n=4/genotype) and female (n=4/genotype) samples in each cell cluster. **(F)** UMAP plot colored by sex demonstrated equivalent representation within each cluster. **(G)** Stacked bar plot showing the normalized proportion of nuclei from each biological sample in each cluster. **(H)** Bar plot showing the number of nuclei for each cell cluster. The dendrogram represents a hierarchical clustering of gene expression values averaged in each cluster for the top 25 cluster marker genes in each cluster, ordering clusters based on transcriptional similarity. **(I)** Heatmap showing additional enriched genes for each cluster. For box and whisker plots in panels **(A)** and **(D)**, box boundaries correspond to the interquartile range (IQR), the line represents the median, and the whiskers extend to the lowest/highest points that are not further than 1.5 times the IQR from the box boundary**.**

Figure S3

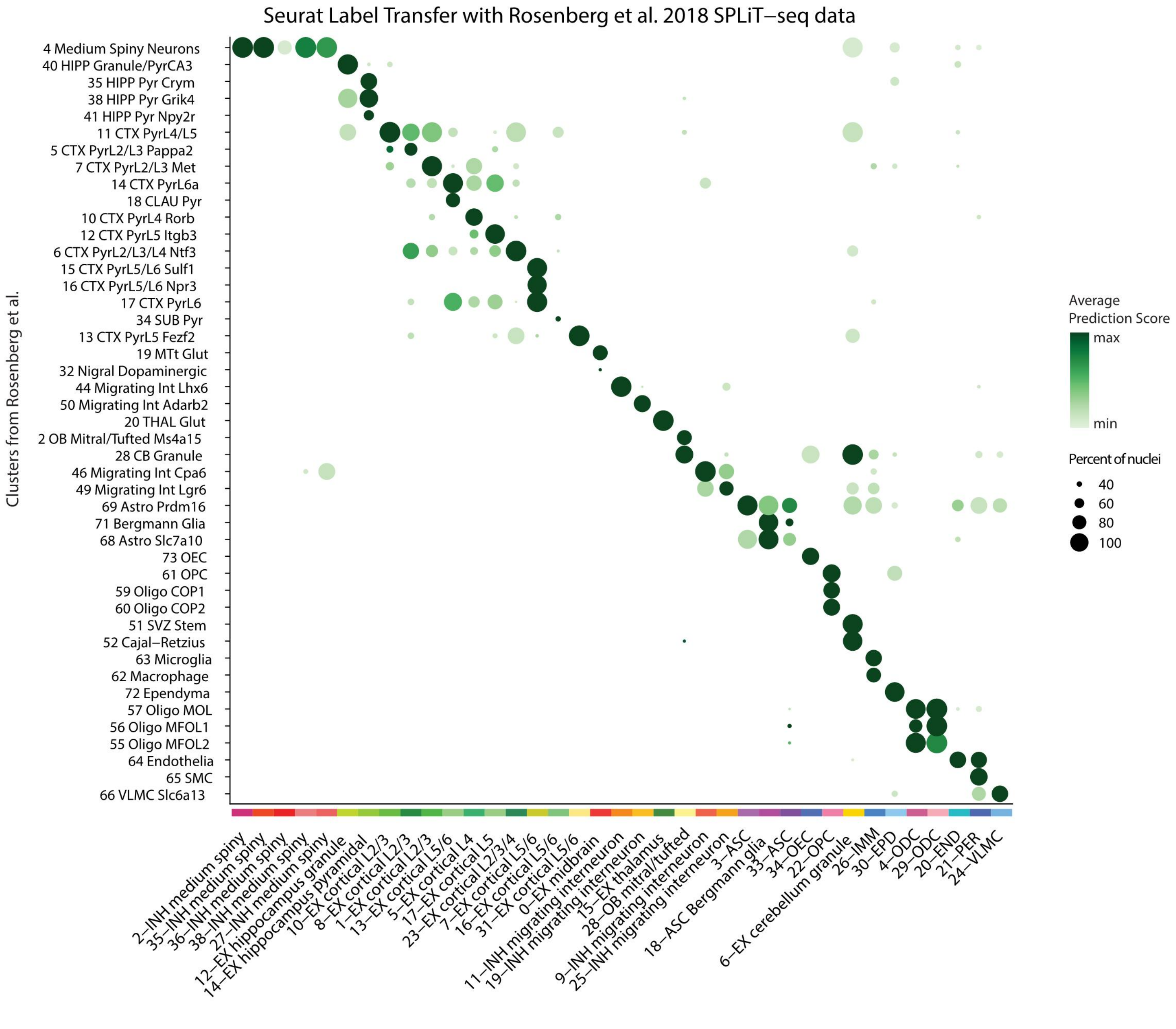
Seurat Label Transfer with Rosenberg et al. 2018 SPLiT−seq data
Clusters from Rosenberg et al.
4 Medium Spiny Neurons
40 HIPP Granule/PyrCA3
35 HIPP Pyr Crym
38 HIPP Pyr Grik4
41 HIPP Pyr Npy2r
11 CTX PyrL4/L5
5 CTX PyrL2/L3 Pappa2
7 CTX PyrL2/L3 Met
14 CTX PyrL6a
18 CLAU Pyr
10 CTX PyrL4 Rorb
12 CTX PyrL5 Itgb3
6 CTX PyrL2/L3/L4 Ntf3
15 CTX PyrL5/L6 Sulf1
16 CTX PyrL5/L6 Npr3
17 CTX PyrL6
34 SUB Pyr
13 CTX PyrL5 Fezf2
19 MTt Glut
32 Nigral Dopaminergic
44 Migrating Int Lhx6
50 Migrating Int Adarb2
20 THAL Glut
2 OB Mitral/Tufted Ms4a15
28 CB Granule
46 Migrating Int Cpa6
49 Migrating Int Lgr6
69 Astro Prdm16
71 Bergmann Glia
68 Astro Slc7a10
73 OEC
61 OPC
59 Oligo COP1
60 Oligo COP2
51 SVZ Stem
52 Cajal−Retzius
63 Microglia
62 Macrophage
72 Ependyma
57 Oligo MOL
56 Oligo MFOL1
55 Oligo MFOL2
64 Endothelia
65 SMC
66 VLMC Slc6a13
2−INH medium spiny
35−INH medium spiny
36−INH medium spiny
38−INH medium spiny
27−INH medium spiny
12−EX hippocampus granule
14−EX hippocampus pyramidal
10−EX cortical L2/3
8−EX cortical L2/3
1−EX cortical L2/3
13−EX cortical L5/6
5−EX cortical L4
17−EX cortical L5
23−EX cortical L2/3/4
7−EX cortical L5/6
16−EX cortical L5/6
31−EX cortical L5/6
0−EX midbrain
11−INH migrating interneuron
19−INH migrating interneuron
15−EX thalamus
28−OB mitral/tufted
9−INH migrating interneuron
25−INH migrating interneuron
3−ASC
18−ASC Bergmann glia
33−ASC
34−OEC
22−OPC
6−EX cerebellum granule
26−IMM
30−EPD
4−ODC
29−ODC
20−END
21−PER
24−VLMC
Average
Prediction Score
max
min
Percent of nuclei
40
60
80
100

**Supplemental Figure 3: Supervised label transfer analysis predicts snRNA-seq cell identity.** Related to Figure 1. Seurat v3 Label Transfer algorithm was used to predict the cell-type identity of each cell in our snRNA-seq dataset based on the annotations and transcriptomic profiles provided in the (Rosenberg et al., 2018) mouse brain snRNA-seq dataset. We report the results in a dot plot where the color of the dot represents the average prediction score in each of our snRNA-seq clusters, and the size of the dot represents the percent of nuclei in each cluster with the predicted score for that cell-type. The horizontal axis shows the different cell annotations from the reference dataset, while the vertical axis shows the cell clusters from the snRNA-seq dataset in the present study.

Figure S4

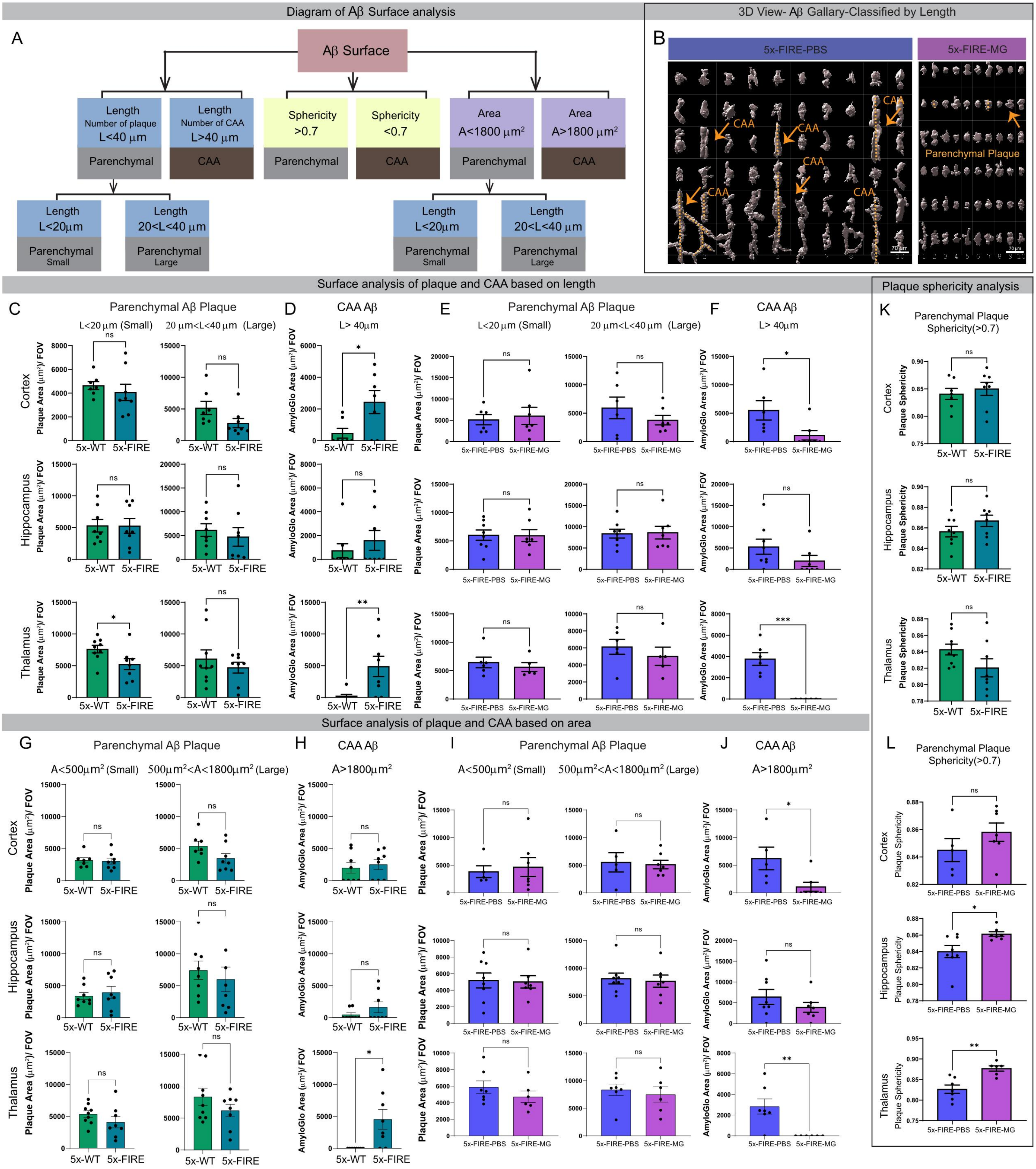

Diagram of Aβ Surface analysis
A
Aβ Surface
Length
Number of plaque
L<40 μm
Parenchymal
Length
Number of CAA
L>40 μm
CAA
Sphericity
>0.7
Parenchymal
Sphericity
<0.7
CAA
Area
A<1800 μm²
Parenchymal
Area
A>1800 μm²
CAA
Length
L<20μm
Parenchymal
Small
Length
20<L<40 μm
Parenchymal
Large
3D View- Aβ Gallary-Classified by Length
B
5x-FIRE-PBS
5x-FIRE-MG
CAA
Parenchymal Plaque
70 μm
Surface analysis of plaque and CAA based on length
C
Parenchymal Aβ Plaque
L<20 μm (Small)
20 μm<L<40 μm (Large)
D
CAA Aβ
L> 40μm
E
Parenchymal Aβ Plaque
L<20 μm (Small)
20 μm<L<40 μm (Large)
F
CAA Aβ
L> 40μm
Plaque sphericity analysis
K
Parenchymal Plaque
Sphericity(>0.7)
Cortex
Hippocampus
Thalamus
Plaque Area (μm²)/ FOV
AmyloGlo Area (μm²)/ FOV
Plaque Sphericity
5x-WT
5x-FIRE
5x-FIRE-PBS
5x-FIRE-MG
ns
*
**
***
Surface analysis of plaque and CAA based on area
G
Parenchymal Aβ Plaque
A<500μm² (Small)
500μm²<A<1800μm² (Large)
H
CAA Aβ
A>1800μm²
I
Parenchymal Aβ Plaque
A<500μm² (Small)
500μm²<A<1800μm² (Large)
J
CAA Aβ
A>1800μm²
L
Parenchymal Plaque
Sphericity(>0.7)

**Supplemental Figure 4: Additional analysis of Ab pathologies.** Related to Figure 2 and 4 **(A)** Schematic of IMARIS plaque and CAA classification. **(B)** Representative IMARIS 3D rendering of CAA morphology in 5x-FIRE-PBS mice and a restoration of parenchymal plaques in 5x-FIRE-MG mice. **(C)** The area occupied by small (<20mm) and large (>20mm and <40mm) parenchymal plaques remains unchanged in 5x-FIRE mice cortex and hippocampus. However, small plaques within the thalamus are reduced in 5x-FIRE mice. **(D)** In contrast, the area occupied by CAA (length ≥40mm) is significantly increased within the cortex and thalamus. **(E)** The area occupied by small and large parenchymal plaques also remains unchanged between 5x-FIRE-PBS and 5x-FIRE-MG groups following microglial transplantation. **(F)** However, microglial transplantation significantly reduced area of CAA in 5x-FIRE-MG mice. **(G-J)** Very similar results are obtained when plaques and CAA are classified in IMARIS based on the area occupied by plaques (<1800mm$^2$) versus CAA (>1800 mm$^2$). Whereas no difference in plaque sphericity is detected between 5x-WT and 5x-FIRE groups **(K)** a significant increase in sphericity is observed within the hippocampus and thalamus following microglia transplantation **(L)**. All data presented as mean ± s.e.m.

Figure S5

Reduction of CAA plaques within 5x-FIRE-MG (Transplanted)

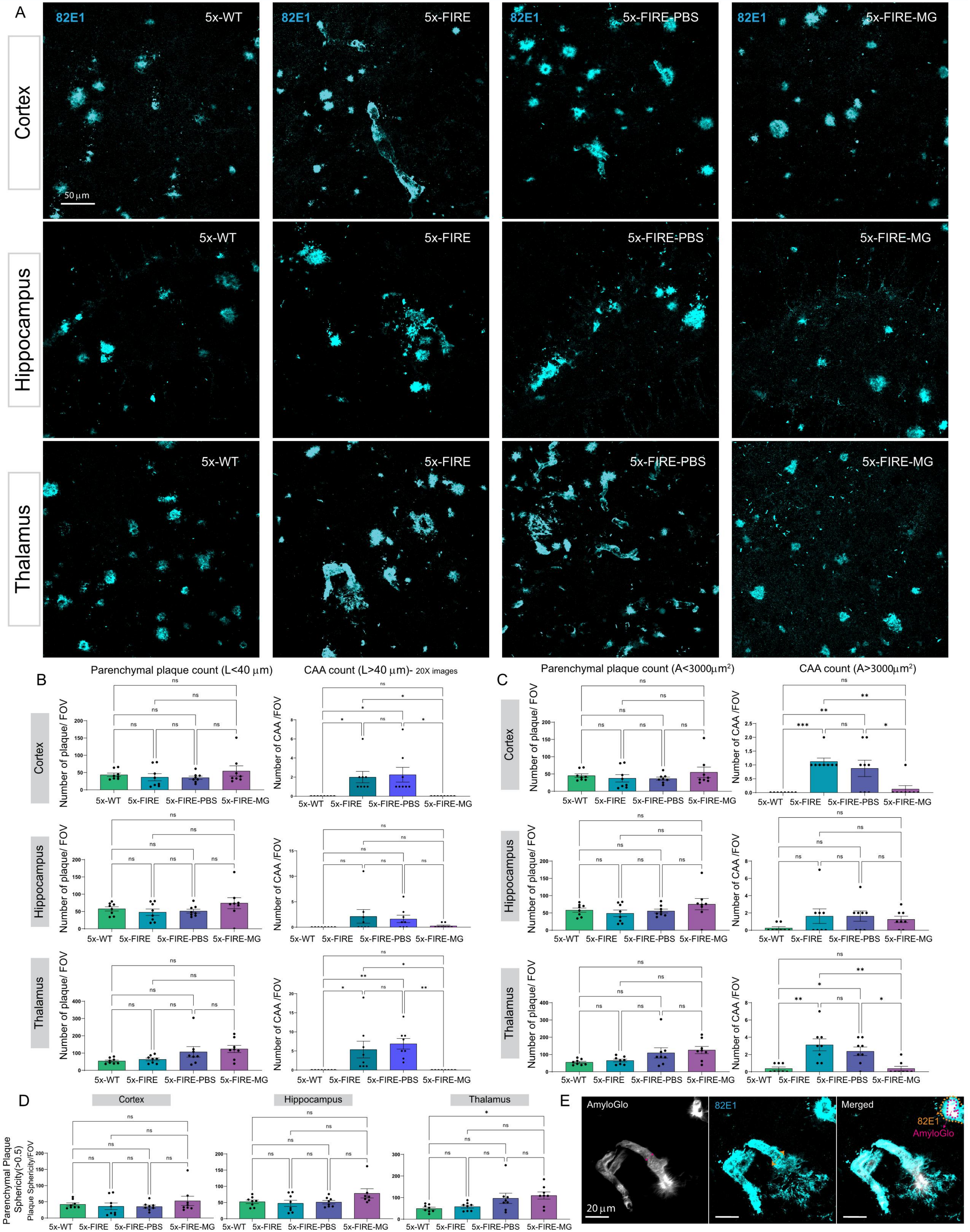

**Supplemental Figure 5: Analysis of beta-amyloid antibody immunoreactivity closely mimics results observed with Amylo-Glo.** Related to Figure 2 and 4. **(A)** As Amylo-Glo labels dense-core plaques more intensely than diffuse plaques, additional analysis of Ab pathology was performed using a biotinylated antibody against human Ab (82E1). **(A)** Confocal images reveal a similar shift in distribution of Ab pathology from parenchymal plaques to CAA in 5x-FIRE mice and a subsequent reversal of these changes in 5x-FIRE-MG mice within the cortex, hippocampus, and thalamus. IMARIS-based quantification of plaque and CAA numbers via either length-based **(B)** or area-based **(C)** classification reveals few changes in plaque numbers but significant increases in CAA that are largely reversed by microglia transplantation. **(D)** Sphericity of plaques is also largely unchanged except within the thalamus where microglial transplantation increases plaque sphericity. **(E)** Co-labeling of 82E1 with Amylo-Glo shows that whereas both approaches sufficiently label CAA, antibody-based labeling detects more diffuse amyloid pathology that surrounds AmyloGlo+ fibrillar deposits. Scale Bars =50mm in A and 20mm in E. All data presented as mean ± s.e.m.

Figure S6

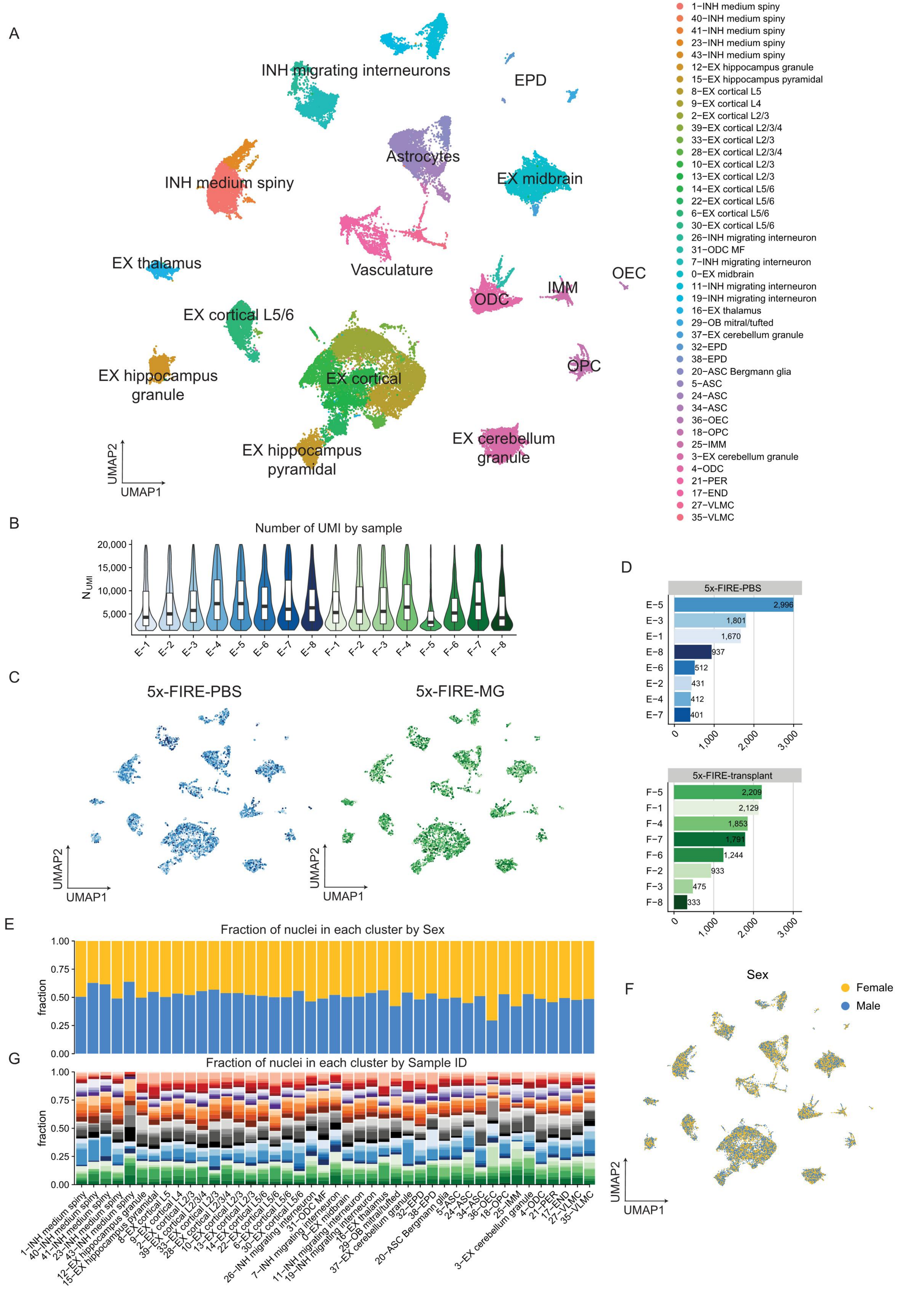
A
INH migrating interneurons
EPD
Astrocytes
INH medium spiny
EX midbrain
EX thalamus
Vasculature
OEC
IMM
ODC
EX cortical L5/6
OPC
EX hippocampus granule
EX cortical
EX cerebellum granule
EX hippocampus pyramidal
UMAP2
UMAP1
1−INH medium spiny
40−INH medium spiny
41−INH medium spiny
23−INH medium spiny
43−INH medium spiny
12−EX hippocampus granule
15−EX hippocampus pyramidal
8−EX cortical L5
9−EX cortical L4
2−EX cortical L2/3
39−EX cortical L2/3/4
33−EX cortical L2/3
28−EX cortical L2/3/4
10−EX cortical L2/3
13−EX cortical L2/3
14−EX cortical L5/6
22−EX cortical L5/6
6−EX cortical L5/6
30−EX cortical L5/6
26−INH migrating interneuron
31−ODC MF
7−INH migrating interneuron
0−EX midbrain
11−INH migrating interneuron
19−INH migrating interneuron
16−EX thalamus
29−OB mitral/tufted
37−EX cerebellum granule
32−EPD
38−EPD
20−ASC Bergmann glia
5−ASC
24−ASC
34−ASC
36−OEC
18−OPC
25−IMM
3−EX cerebellum granule
4−ODC
21−PER
17−END
27−VLMC
35−VLMC
B
Number of UMI by sample
20,000
15,000
10,000
5,000
N UMI
E−1 E−2 E−3 E−4 E−5 E−6 E−7 E−8 F−1 F−2 F−3 F−4 F−5 F−6 F−7 F−8
C
5x-FIRE-PBS
5x-FIRE-MG
UMAP2
UMAP1
D
5x-FIRE-PBS
E−5 2,996
E−3 1,801
E−1 1,670
E−8 937
E−6 512
E−2 431
E−4 412
E−7 401
0 1,000 2,000 3,000
5x-FIRE-transplant
F−5 2,209
F−1 2,129
F−4 1,853
F−7 1,791
F−6 1,244
F−2 933
F−3 475
F−8 333
0 1,000 2,000 3,000
E
Fraction of nuclei in each cluster by Sex
1.00
0.75
0.50
0.25
0.00
fraction
F
Sex
Female
Male
UMAP2
UMAP1
G
Fraction of nuclei in each cluster by Sample ID
1.00
0.75
0.50
0.25
0.00
fraction
1−INH medium spiny
40−INH medium spiny
41−INH medium spiny
23−INH medium spiny
43−INH medium spiny
12−EX hippocampus granule
15−EX hippocampus pyramidal
8−EX cortical L5
9−EX cortical L4
2−EX cortical L2/3
39−EX cortical L2/3/4
33−EX cortical L2/3
28−EX cortical L2/3/4
10−EX cortical L2/3
13−EX cortical L2/3
14−EX cortical L5/6
22−EX cortical L5/6
6−EX cortical L5/6
30−EX cortical L5/6
26−INH migrating interneuron
31−ODC MF
7−INH migrating interneuron
0−EX midbrain
11−INH migrating interneuron
19−INH migrating interneuron
16−EX thalamus
29−OB mitral/tufted
37−EX cerebellum granule
32−EPD
38−EPD
20−ASC Bergmann glia
5−ASC
24−ASC
34−ASC
36−OEC
18−OPC
25−IMM
3−EX cerebellum granule
4−ODC
21−PER
17−END
27−VLMC
35−VLMC

**Supplemental Figure 6: snRNA-seq analysis including the 5xFIRE transplanted microglia condition.** Related to Figure 5. **(A)** UMAP plot colored by leiden cluster assignment for 43 clusters. Major cell lineages are annotated directly on the plot. **(B)** Violin plots showing the distribution of the number of UMIs captured in each biological sample for the 5xFIRE-PBS and 5xFIRE-MG conditions. Box boundaries correspond to the interquartile range (IQR), the line represents the median, and the whiskers extend to the lowest/highest points that are not further than 1.5 times the IQR from the box boundary. **(C)** UMAP plots as in (A) subset by nuclei with from 5xFIRE-PBS (left) and 5xFIRE-MG (right) conditions, colored by biological sample of origin. **(D)** Bar plot reporting the number of nuclei recovered in each sample from the 5xFIRE-PBS and 5xFIRE-MG groups after quality-control filtering. **(E)** Stacked bar plot showing the normalized proportion of nuclei from male and female samples in each cell cluster. **(F)** UMAP plot as in (A) where cells are colored by sex. **(G)** Stacked bar plot showing the normalized proportion of nuclei from each biological sample in each cluster.

Figure S7

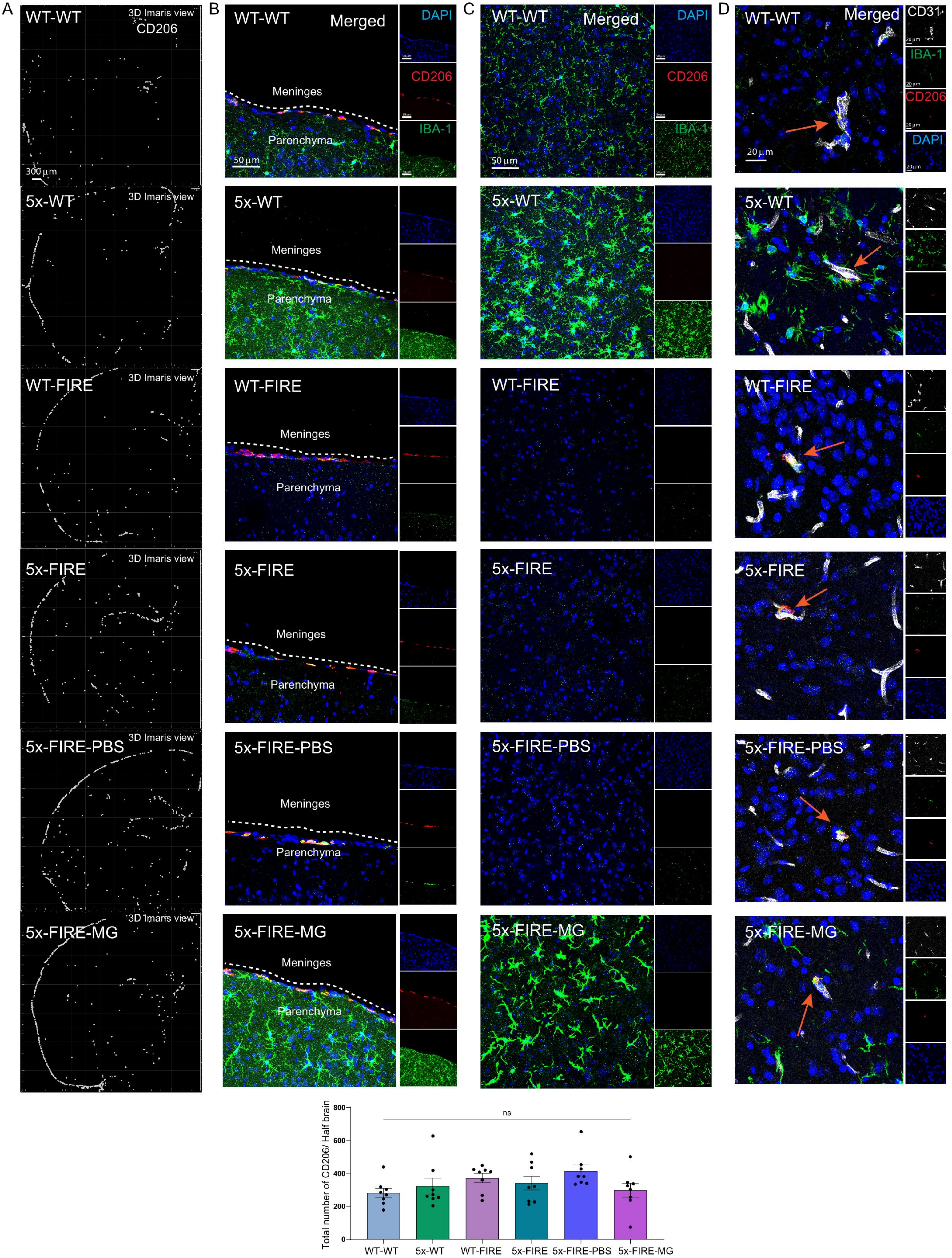
A
B
C
D
WT-WT
5x-WT
WT-FIRE
5x-FIRE
5x-FIRE-PBS
5x-FIRE-MG
3D Imaris view
CD206
300 μm
Merged
DAPI
CD206
IBA-1
CD31
Meninges
Parenchyma
50 μm
20 μm
ns
Total number of CD206/ Half brain
0
200
400
600
800

**Supplemental Figure 7: Meningeal and perivascular macrophages remain unchanged in FIRE mice.** Related to Figure 1, 2, and 3. **(A)** Although previous studied detected no changes in meningeal and perivascular macrophage populations in FIRE mice it is possible that the additional impact of beta-amyloid pathology could alter these populations. Therefore, CD206 immunolabeling was used to detect and quantify these populations in each of the six groups of mice. **(A)** Low power stiches from confocal Z-stack images were examined by IMARIS spot detection and white dots placed to depict CD206+ cells. Co-labeling with DAPI (blue), CD206 (red), and IBA-1 (green) illustrates the localization of CD206+ meningeal macrophages **(B)** and a lack of CD206 staining within parenchymal microglia **(C)**. CD206+ cells are also occasionally observed directly adjacent to CD31+ blood vessel endothelial cells **(D)** demonstrating the detection of perivascular macrophages. **(E)** Quantification of total CD206+ cells within the brains of each genotype reveal no significant differences. Scale Bars =300mm in A, 50mm in B and C, 20mm in D. All data presented as mean ± s.e.m.

Figure S8

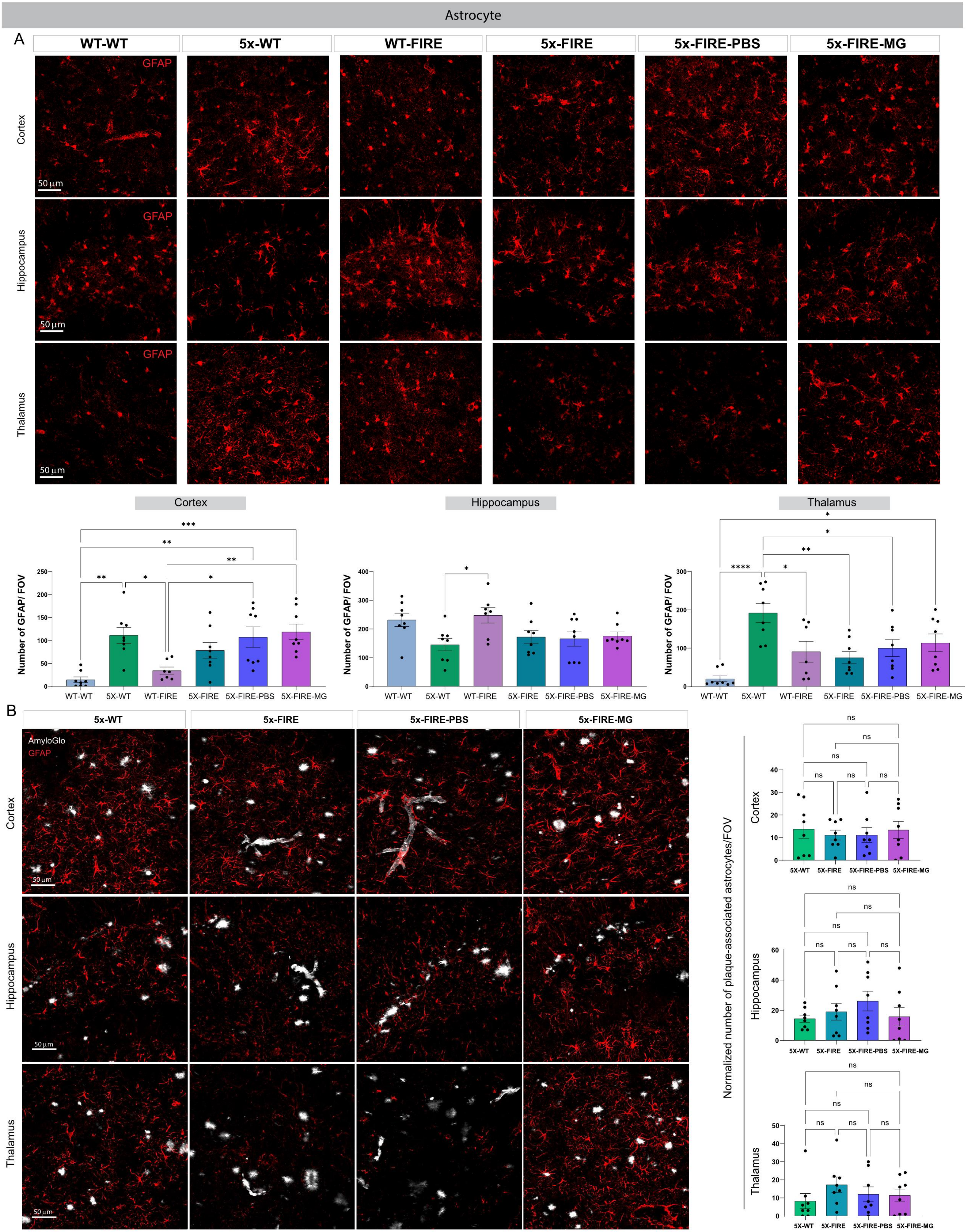
Astrocyte
A
WT-WT
5x-WT
WT-FIRE
5x-FIRE
5x-FIRE-PBS
5x-FIRE-MG
GFAP
Cortex
Hippocampus
Thalamus
50 μm
Cortex
Hippocampus
Thalamus
Number of GFAP/ FOV
WT-WT
5X-WT
WT-FIRE
5X-FIRE
5X-FIRE-PBS
5X-FIRE-MG
B
5x-WT
5x-FIRE
5x-FIRE-PBS
5x-FIRE-MG
AmyloGlo
GFAP
Normalized number of plaque-associated astrocytes/FOV
ns
5X-WT
5X-FIRE
5X-FIRE-PBS
5X-FIRE-MG

**Supplemental Figure 8: Few changes in astrocyte numbers are detected in 5x-FIRE mice.** Related to Figure 2 and 4. To determine whether the absence of microglia in FIRE mice impacts the number or responsiveness of astrocytes to Ab plaques we examined GFAP immunoreactivity. **(A)** GFAP immunoreactivity within the cortex, hippocampus, and thalamus of all six groups of mice is shown. Quantification revealed a significant increase in GFAP+ astrocytes within the cortex and thalamus of 5x-WT mice versus WT-WT mice but few differences between 5x-WT and 5x-FIRE groups. However, astrocyte numbers within the thalamus were significantly reduced in 5x-FIRE versus 5x-WT mice and partially restored following microglial transplantation. **(B)** To determine whether 5x-FIRE mice exhibit an altered association between astrocytes and Ab pathology proximity of astrocytes to Ab plaques were quantified but revealed no significant differences. Scale Bars =50mm in A and B. All data presented as mean ± s.e.m.

Figure S9

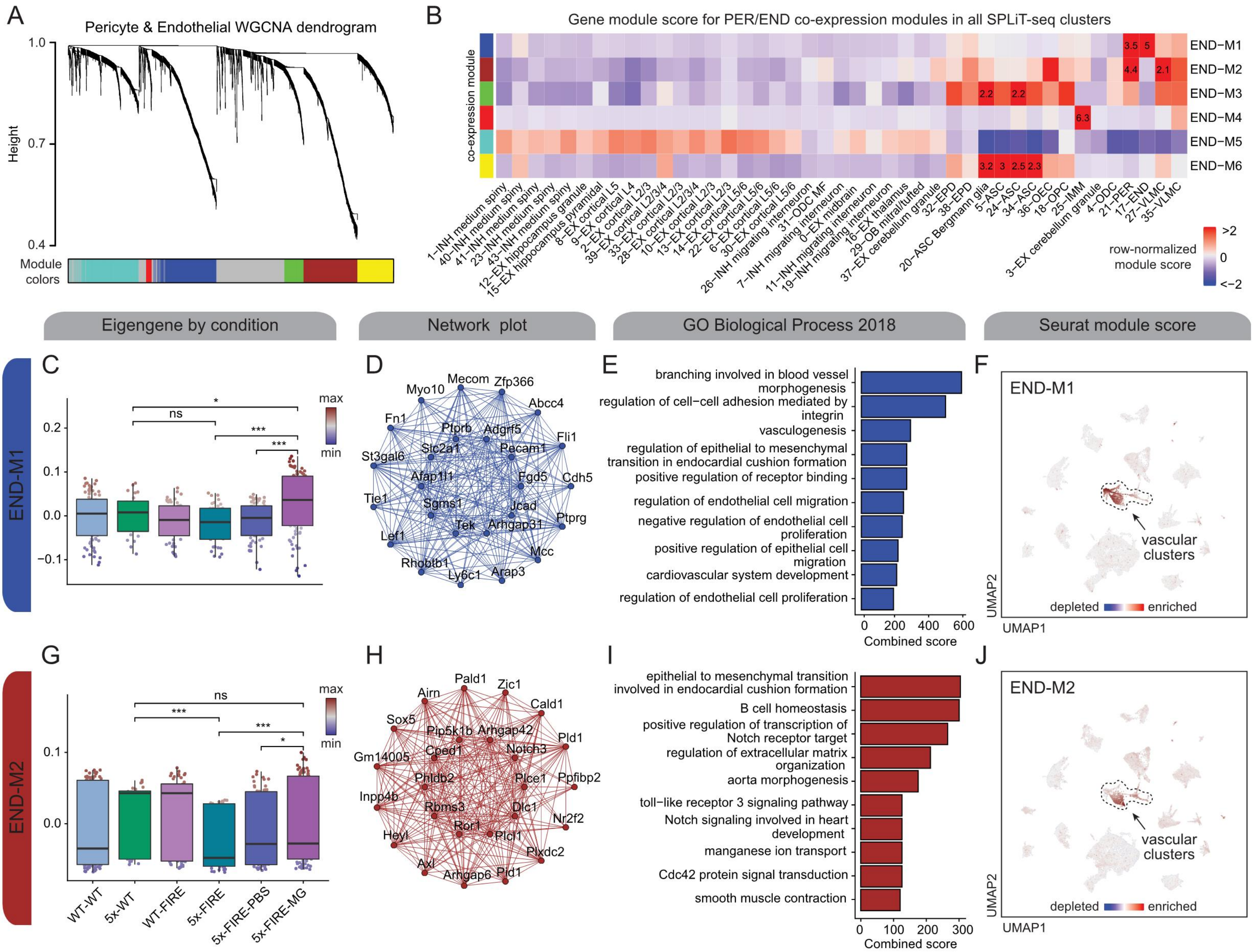
A
Pericyte & Endothelial WGCNA dendrogram
Height
Module colors
B
Gene module score for PER/END co-expression modules in all SPLiT-seq clusters
co-expression module
END-M1
END-M2
END-M3
END-M4
END-M5
END-M6
row-normalized module score
Eigengene by condition
Network plot
GO Biological Process 2018
Seurat module score
END-M1
END-M2
branching involved in blood vessel morphogenesis
regulation of cell−cell adhesion mediated by integrin
vasculogenesis
regulation of epithelial to mesenchymal transition in endocardial cushion formation
positive regulation of receptor binding
regulation of endothelial cell migration
negative regulation of endothelial cell proliferation
positive regulation of epithelial cell migration
cardiovascular system development
regulation of endothelial cell proliferation
Combined score
epithelial to mesenchymal transition involved in endocardial cushion formation
B cell homeostasis
positive regulation of transcription of Notch receptor target
regulation of extracellular matrix organization
aorta morphogenesis
toll−like receptor 3 signaling pathway
Notch signaling involved in heart development
manganese ion transport
Cdc42 protein signal transduction
smooth muscle contraction
vascular clusters
depleted
enriched
UMAP1
UMAP2
WT-WT
5x-WT
WT-FIRE
5x-FIRE
5x-FIRE-PBS
5x-FIRE-MG

**Supplemental Figure 9: Gene co-expression networks in endothelial cells and pericytes.** Related to Figure 1, 3, 5, and 6. **(A)** scWGCNA dendrogram showing co-expression modules in endothelial cells and pericytes (END and PER) with gene module assignments shown below. **(B)** Row-normalized heatmap of Seurat module scores by SPLiT-seq clusters for all PER/END WGCNA modules as in (A). **(C)** Box and whisker plots with underlying data points for END-M1 eigengene values split by experimental condition. Box boundaries correspond to the interquartile range (IQR), the line represents the median, and the whiskers extend to the lowest/highest points that are not further than 1.5 times the IQR from the box boundary. **(D)** Co-expression network plot showing the 25 most connected genes for END-M1. **(E)** Bar plot showing GO term enrichment for END-M1 genes. **(F)** UMAP colored by Seurat module score for END-M1 with END and PER clusters circled. **(G-J):** Eigengene box and whisker plot, co-expression network plot, GO term enrichment, and module score UMAP as in (C-F) for END-M2. For box and whisker plots, two-sided Wilcoxon test was used to compare selected groups; not significant, $P > 0.05$; $*P \leq 0.05$, $**P \leq 0.01$, $***P \leq 0.001$, $****P \leq 0.0001$.

Figure S10

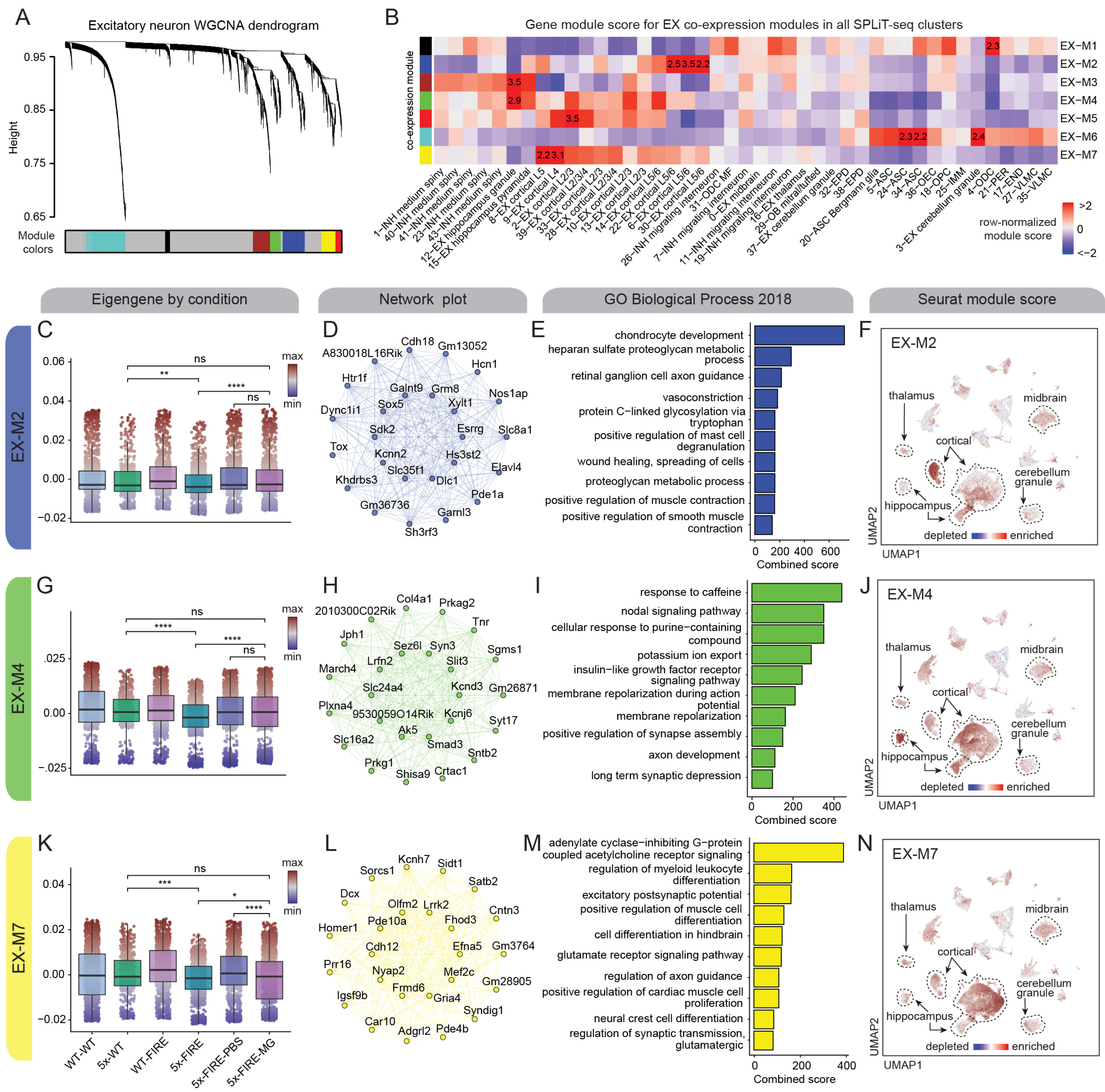
A
Excitatory neuron WGCNA dendrogram
Height
Module colors
B
Gene module score for EX co-expression modules in all SPLiT-seq clusters
co-expression module
EX-M1
EX-M2
EX-M3
EX-M4
EX-M5
EX-M6
EX-M7
row-normalized module score
Eigengene by condition
Network plot
GO Biological Process 2018
Seurat module score
EX-M2
chondrocyte development
heparan sulfate proteoglycan metabolic process
retinal ganglion cell axon guidance
vasoconstriction
protein C−linked glycosylation via tryptophan
positive regulation of mast cell degranulation
wound healing, spreading of cells
proteoglycan metabolic process
positive regulation of muscle contraction
positive regulation of smooth muscle contraction
Combined score
thalamus
midbrain
cortical
cerebellum granule
hippocampus
depleted
enriched
UMAP1
UMAP2
EX-M4
response to caffeine
nodal signaling pathway
cellular response to purine−containing compound
potassium ion export
insulin−like growth factor receptor signaling pathway
membrane repolarization during action potential
membrane repolarization
positive regulation of synapse assembly
axon development
long term synaptic depression
EX-M7
adenylate cyclase−inhibiting G−protein coupled acetylcholine receptor signaling
regulation of myeloid leukocyte differentiation
excitatory postsynaptic potential
positive regulation of muscle cell differentiation
cell differentiation in hindbrain
glutamate receptor signaling pathway
regulation of axon guidance
positive regulation of cardiac muscle cell proliferation
neural crest cell differentiation
regulation of synaptic transmission, glutamatergic
WT-WT
5x-WT
WT-FIRE
5x-FIRE
5x-FIRE-PBS
5x-FIRE-MG

**Supplemental Figure 10: Gene co-expression networks in excitatory neurons.** Related to Figure 1, 3, 5, and 6. **(A)** scWGCNA dendrogram showing co-expression modules in excitatory neurons with gene module assignments shown below. **(B)** Row-normalized heatmap of Seurat module scores by SPLiT-seq clusters for all EX WGCNA modules as in **(A,C)**: Box and whisker plots with underlying data points for EX-M2 eigengene values split by experimental condition. Box boundaries correspond to the interquartile range (IQR), the line represents the median, and the whiskers extend to the lowest/highest points that are not further than 1.5 times the IQR from the box boundary. **(D)** Co-expression network plot showing the 25 most connected genes for EX-M2. **(E)** Bar plot showing GO term enrichment for EX-M2 genes. **(F)** UMAP colored by Seurat module score for EX-M2 with astrocytes circled. **(G-J)** Eigengene box and whisker plot, co-expression network plot, GO term enrichment, and module score UMAP as in **C-F** for EX-M4. **(K-N):** Eigengene box and whisker plot, co-expression network plot, GO term enrichment, and module score UMAP as in (C-F) for EX-M7. For box and whisker plots, two-sided Wilcoxon test was used to compare selected groups; not significant, $P > 0.05$; $^*P \leq 0.05$, $^{**}P \leq 0.01$, $^{***}P \leq 0.001$, $^{****}P \leq 0.0001$.

Figure S11

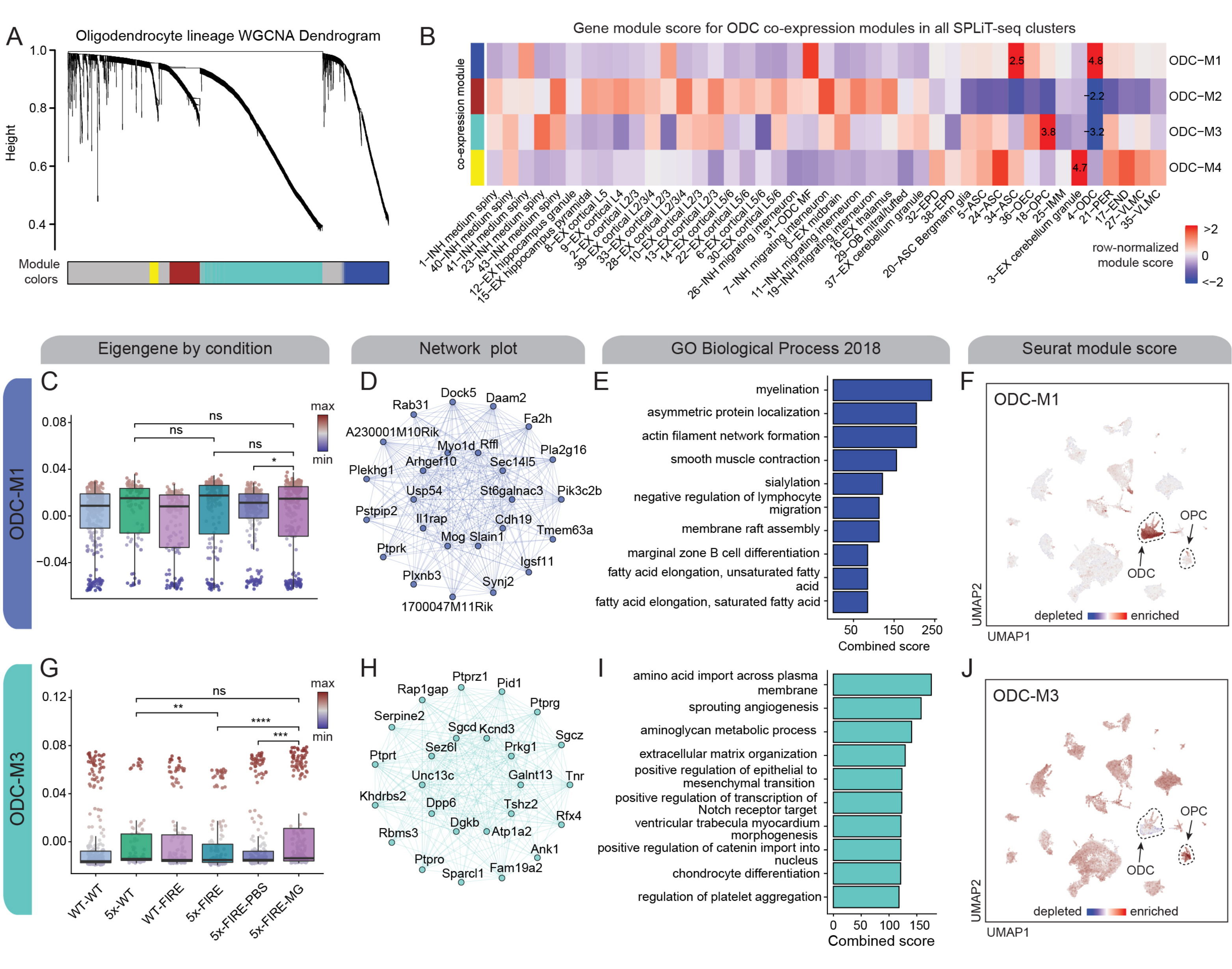

A
Oligodendrocyte lineage WGCNA Dendrogram
Height
Module colors
B
Gene module score for ODC co-expression modules in all SPLiT-seq clusters
co-expression module
ODC−M1
ODC−M2
ODC−M3
ODC−M4
row-normalized module score
>2
0
<−2
Eigengene by condition
Network plot
GO Biological Process 2018
Seurat module score
C
ODC-M1
D
E
myelination
asymmetric protein localization
actin filament network formation
smooth muscle contraction
sialylation
negative regulation of lymphocyte migration
membrane raft assembly
marginal zone B cell differentiation
fatty acid elongation, unsaturated fatty acid
fatty acid elongation, saturated fatty acid
Combined score
F
ODC-M1
OPC
ODC
depleted
enriched
UMAP2
UMAP1
G
ODC-M3
WT-WT
5x-WT
WT-FIRE
5x-FIRE
5x-FIRE-PBS
5x-FIRE-MG
H
I
amino acid import across plasma membrane
sprouting angiogenesis
aminoglycan metabolic process
extracellular matrix organization
positive regulation of epithelial to mesenchymal transition
positive regulation of transcription of Notch receptor target
ventricular trabecula myocardium morphogenesis
positive regulation of catenin import into nucleus
chondrocyte differentiation
regulation of platelet aggregation
Combined score
J
ODC-M3
OPC
ODC
depleted
enriched
UMAP2
UMAP1

**Supplemental Figure 11: Gene co-expression networks in the oligodendrocyte lineage.** Related to Figure 1, 3, 5, and 6. **(A)** scWGCNA dendrogram showing co-expression modules in oligodendrocyte lineage cells (ODCs and OPCs) with gene module assignments shown below. **(B)**Row-normalized heatmap of Seurat module scores by SPLiT-seq clusters for all ODC WGCNA modules as in **(A, C)** Box and whisker plots with underlying data points for ODC-M1 eigengene values split by experimental condition. Box boundaries correspond to the interquartile range (IQR), the line represents the median, and the whiskers extend to the lowest/highest points that are not further than 1.5 times the IQR from the box boundary. **(D)** Co-expression network plot showing the 25 most connected genes for ODC-M1. **(E)** Bar plot showing GO term enrichment for ODC-M1 genes. **(F)** UMAP colored by Seurat module score for ODC-M1 with ODCs and OPCs circled. **(G-J)** Eigengene box and whisker plot, co-expression network plot, GO term enrichment, and module score UMAP as in (C-F) for ODC-M3. For box and whisker plots, two-sided Wilcoxon test was used to compare selected groups; not significant, $P > 0.05$; $^*P \leq 0.05$, $^{**}P \leq 0.01$, $^{***}P \leq 0.001$, $^{****}P \leq 0.0001$.

Figure S12

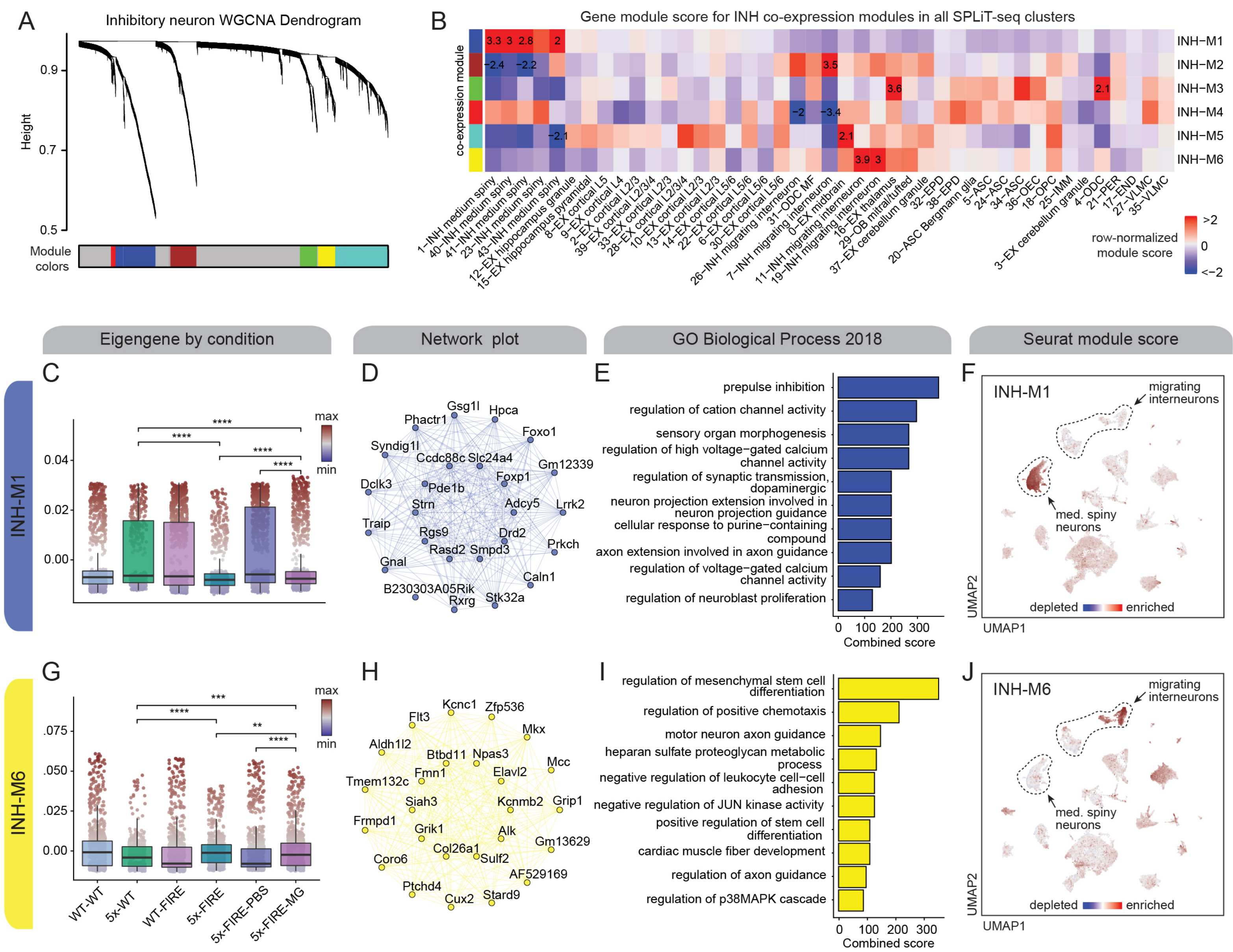
A
Inhibitory neuron WGCNA Dendrogram
Height
Module colors
B
Gene module score for INH co-expression modules in all SPLiT-seq clusters
co-expression module
INH−M1
INH−M2
INH−M3
INH−M4
INH−M5
INH−M6
row-normalized module score
Eigengene by condition
Network plot
GO Biological Process 2018
Seurat module score
C
INH-M1
max
min
D
Gsg1l
Hpca
Phactr1
Foxo1
Syndig1l
Ccdc88c
Slc24a4
Gm12339
Dclk3
Pde1b
Foxp1
Strn
Adcy5
Lrrk2
Traip
Rgs9
Drd2
Rasd2
Smpd3
Prkch
Gnal
Caln1
B230303A05Rik
Rxrg
Stk32a
E
prepulse inhibition
regulation of cation channel activity
sensory organ morphogenesis
regulation of high voltage−gated calcium channel activity
regulation of synaptic transmission, dopaminergic
neuron projection extension involved in neuron projection guidance
cellular response to purine−containing compound
axon extension involved in axon guidance
regulation of voltage−gated calcium channel activity
regulation of neuroblast proliferation
Combined score
F
INH-M1
migrating interneurons
med. spiny neurons
depleted
enriched
UMAP1
UMAP2
G
INH-M6
max
min
WT-WT
5x-WT
WT-FIRE
5x-FIRE
5x-FIRE-PBS
5x-FIRE-MG
H
Kcnc1
Zfp536
Flt3
Mkx
Aldh1l2
Btbd11
Npas3
Mcc
Fmn1
Elavl2
Tmem132c
Siah3
Kcnmb2
Grip1
Frmpd1
Grik1
Alk
Gm13629
Col26a1
Sulf2
Coro6
AF529169
Ptchd4
Stard9
Cux2
I
regulation of mesenchymal stem cell differentiation
regulation of positive chemotaxis
motor neuron axon guidance
heparan sulfate proteoglycan metabolic process
negative regulation of leukocyte cell−cell adhesion
negative regulation of JUN kinase activity
positive regulation of stem cell differentiation
cardiac muscle fiber development
regulation of axon guidance
regulation of p38MAPK cascade
Combined score
J
INH-M6
migrating interneurons
med. spiny neurons
depleted
enriched
UMAP1
UMAP2

**Supplemental Figure 12: Gene co-expression networks in inhibitory neurons.** Related to Figure 1, 3, 5, and 6. **(A)** scWGCNA dendrogram showing co-expression modules in inhibitory neurons with gene module assignments shown below. **(B)** Row-normalized heatmap of Seurat module scores by SPLiT-seq clusters for all INH WGCNA modules as in (**A, C)** Box and whisker plots with underlying data points for INH-M1 eigengene values split by experimental condition. Box boundaries correspond to the interquartile range (IQR), the line represents the median, and the whiskers extend to the lowest/highest points that are not further than 1.5 times the IQR from the box boundary. **(D)** Co-expression network plot showing the 25 most connected genes for INH-M1. **(E)** Bar plot showing GO term enrichment for INH-M1 genes. **(F)** UMAP colored by Seurat module score for INH-M1 with END and PER clusters circled. **(G-J)** Eigengene box and whisker plot, co-expression network plot, GO term enrichment, and module score UMAP as in (C-F) for INH-M6. For box and whisker plots, two-sided Wilcoxon test was used to compare selected groups; not significant, $P > 0.05$; $*P \leq 0.05$, $**P \leq 0.01$, $***P \leq 0.001$, $****P \leq 0.0001$.

Figure S13

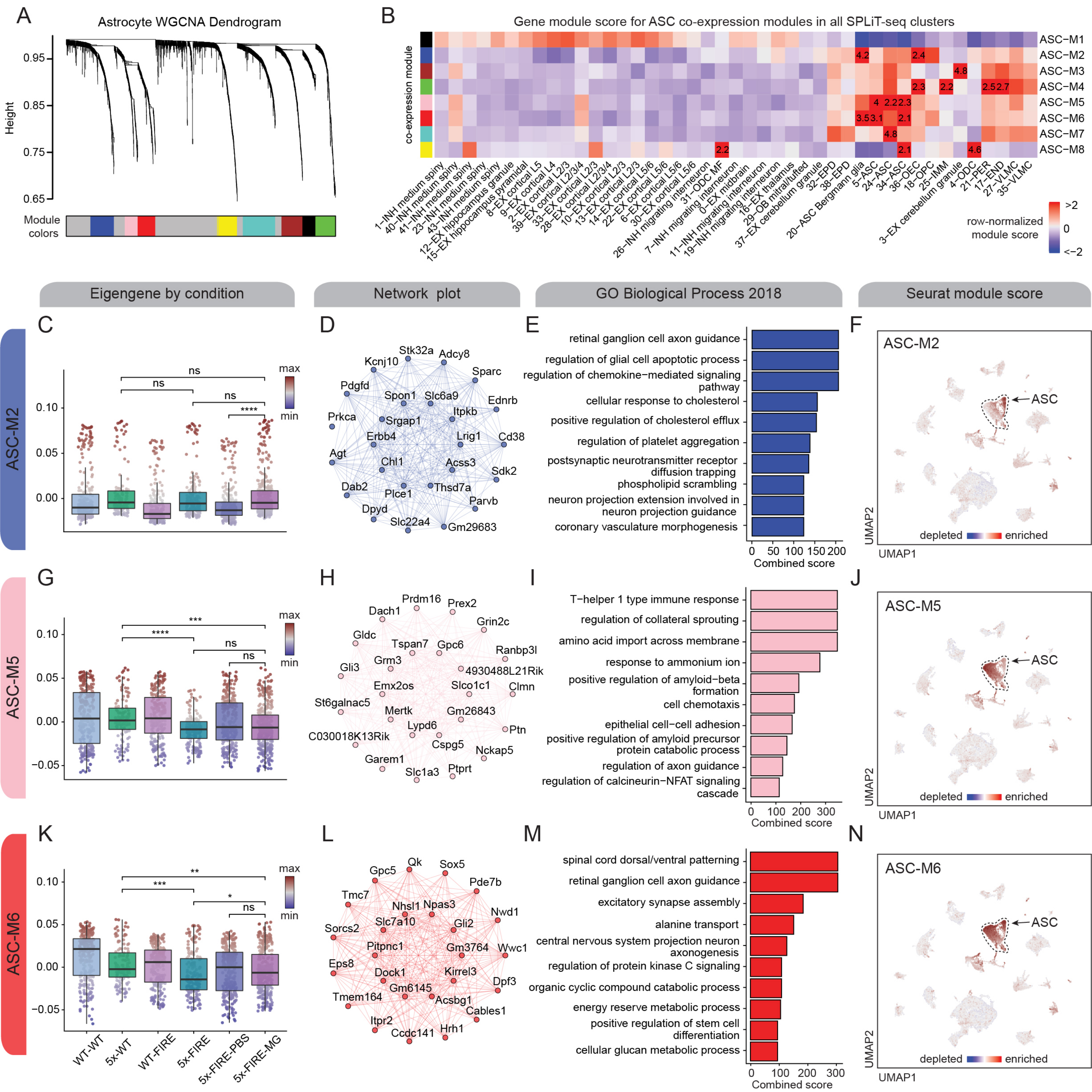

A
Astrocyte WGCNA Dendrogram
Height
Module colors
B
Gene module score for ASC co-expression modules in all SPLiT-seq clusters
co-expression module
ASC−M1
ASC−M2
ASC−M3
ASC−M4
ASC−M5
ASC−M6
ASC−M7
ASC−M8
row-normalized module score
Eigengene by condition
Network plot
GO Biological Process 2018
Seurat module score
C
D
E
F
ASC-M2
retinal ganglion cell axon guidance
regulation of glial cell apoptotic process
regulation of chemokine−mediated signaling pathway
cellular response to cholesterol
positive regulation of cholesterol efflux
regulation of platelet aggregation
postsynaptic neurotransmitter receptor diffusion trapping
phospholipid scrambling
neuron projection extension involved in neuron projection guidance
coronary vasculature morphogenesis
Combined score
depleted
enriched
UMAP1
UMAP2
G
H
I
J
ASC-M5
T−helper 1 type immune response
regulation of collateral sprouting
amino acid import across membrane
response to ammonium ion
positive regulation of amyloid−beta formation
cell chemotaxis
epithelial cell−cell adhesion
positive regulation of amyloid precursor protein catabolic process
regulation of axon guidance
regulation of calcineurin−NFAT signaling cascade
K
L
M
N
ASC-M6
spinal cord dorsal/ventral patterning
retinal ganglion cell axon guidance
excitatory synapse assembly
alanine transport
central nervous system projection neuron axonogenesis
regulation of protein kinase C signaling
organic cyclic compound catabolic process
energy reserve metabolic process
positive regulation of stem cell differentiation
cellular glucan metabolic process
WT-WT
5x-WT
WT-FIRE
5x-FIRE
5x-FIRE-PBS
5x-FIRE-MG
ASC

**Supplemental Figure 13: Gene co-expression networks in astrocytes.** Related to Figure 1, 3, 5, and 6. **(A)** scWGCNA dendrogram showing co-expression modules in astrocytes with gene module assignments shown below. **(B)** Row-normalized heatmap of Seurat module scores by SPLiT-seq clusters for all ASC WGCNA modules as in **(A, C)** Box and whisker plots with underlying data points for ASC-M2 eigengene values split by experimental condition. Box boundaries correspond to the interquartile range (IQR), the line represents the median, and the whiskers extend to the lowest/highest points that are not further than 1.5 times the IQR from the box boundary. **(D)** Co-expression network plot showing the 25 most connected genes for ASC-M2. **(E)** Bar plot showing GO term enrichment for ASC-M2 genes. **(F)** UMAP colored by Seurat module score for ASC-M2 with astrocytes circled. **(G-J)** Eigengene box and whisker plot, co-expression network plot, GO term enrichment, and module score UMAP as in (C-F) for ASC-M5. **(K-N)** Eigengene box and whisker plot, co-expression network plot, GO term enrichment, and module score UMAP as in (C-F) for ASC-M6. For box and whisker plots, two-sided Wilcoxon test was used to compare selected groups; not significant, $P > 0.05$; $*P \leq 0.05$, $**P \leq 0.01$, $***P \leq 0.001$, $****P \leq 0.0001$.

Figure S14

No indication of cerebral hemorrhages or brain calcification in 2-month-old mice

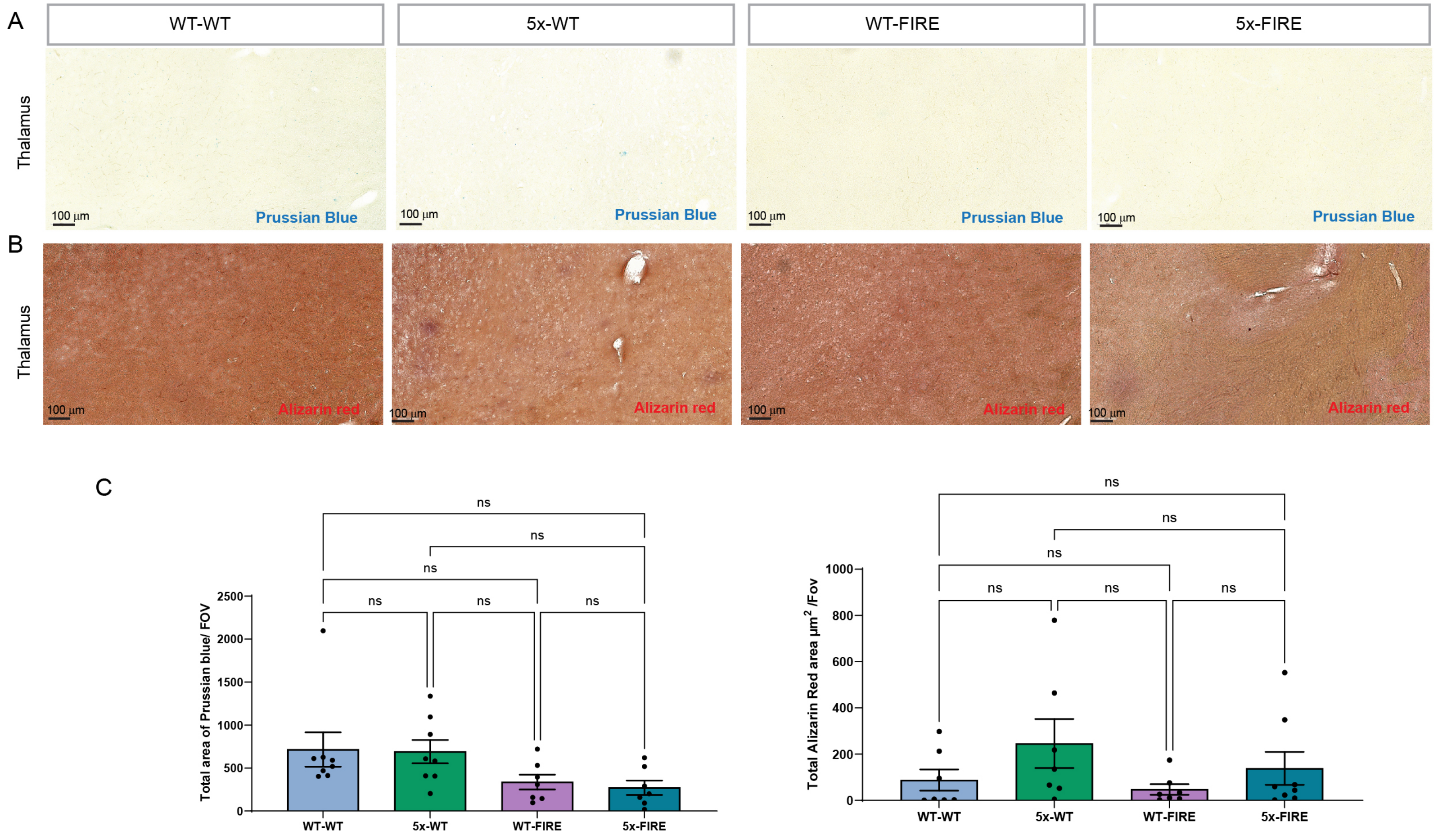

**Supplemental Figure 14: 2-month-old WT-FIRE and 5x-FIRE mice show no evidence of cerebral hemorrhages or brain calcification.** Related to Figure 5, 6 and 7. To determine whether the previously observed effects of combined amyloid pathology and microglial absence on brain hemorrhages and calcification might arise during development we examined young adult mice (2-month-old) from each of the four core genotypes. **(A)** Prussian Blue labeling (thalamus shown) revealed no evidence of blood brain barrier dysfunction or cerebral hemorrhages in either WT-WT, 5x-WT, WT-FIRE, or 5x-FIRE mice. **(B)** No evidence of Alizarin Red labeling was detected within the thalamus (shown) or any other brain region of WT-WT, 5x-WT, WT-FIRE, or 5x-FIRE mice. **(C)** Quantification of Prussian Blue and Alizarin Red labeling revealed no significant increase in signal intensity above WT-WT background levels. Scale Bars =100mm in A and B. All data presented as mean ± s.e.m.
